# Sprouty2 positively regulates T cell function and airway inflammation through regulation of CSK and LCK kinases

**DOI:** 10.1371/journal.pbio.3001063

**Published:** 2021-03-08

**Authors:** Anand Sripada, Kapil Sirohi, Lidia Michalec, Lei Guo, Jerome T. McKay, Sangya Yadav, Mukesh Verma, James Good, Donald Rollins, Magdalena M. Gorska, Rafeul Alam

**Affiliations:** 1 Division of Allergy and Immunology, Department of Medicine, National Jewish Health, Denver, Colorado, United States of America; 2 Division of Pulmonary and Critical Care, Department of Medicine, National Jewish Health, Denver, Colorado, United States of America; 3 Department of Medicine, University of Colorado Anschutz Medical Campus, Aurora, Colorado, United States of America; National Cancer Institute, UNITED STATES

## Abstract

The function of Sprouty2 (Spry2) in T cells is unknown. Using 2 different (inducible and T cell–targeted) knockout mouse strains, we found that Spry2 positively regulated extracellular signal-regulated kinase 1/2 (ERK1/2) signaling by modulating the activity of LCK. Spry2^−/−^ CD4^+^ T cells were unable to activate LCK, proliferate, differentiate into T helper cells, or produce cytokines. Spry2 deficiency abrogated type 2 inflammation and airway hyperreactivity in a murine model of asthma. Spry2 expression was higher in blood and airway CD4^+^ T cells from patients with asthma, and Spry2 knockdown impaired human T cell proliferation and cytokine production. Spry2 deficiency up-regulated the lipid raft protein caveolin-1, enhanced its interaction with CSK, and increased CSK interaction with LCK, culminating in augmented inhibitory phosphorylation of LCK. Knockdown of CSK or dislodgment of caveolin-1–bound CSK restored ERK1/2 activation in Spry2^−/−^ T cells, suggesting an essential role for Spry2 in LCK activation and T cell function.

## Abstract 2

The ubiquitously expressed protein Sprouty2 (Spry2) is a known regulator of Ras/ERK signaling pathway. This study uses in vitro and in vivo models, as well as human specimens, to reveal a mechanism for Spry2 in positively regulating ERK activation in CD4+ T-cells.

## Introduction

Since its discovery as an inhibitor of fibroblast growth factor (FGF)-stimulated tubular morphogenesis [1], Sprouty (Spry) proteins have been implicated in numerous biological processes including limb development [2], lens morphogenesis [3], inner ear development [4], and kidney development [5] (reviewed in [6,7]). To date, 4 mammalian Spry orthologs (Spry1 to 4) have been identified [1,8], and their inhibitory function is both cell and ligand specific. While the expression of Spry3 is restricted to brain and testis, the rest of the Spry proteins are ubiquitously expressed and have been shown to inhibit the Ras/ERK1/2 signaling pathway [9,10]. This general inhibitory function of Spry is due to its ability to interact with key signaling molecules in the Ras/ERK1/2 signaling pathway such as Grb2, Shp2, Raf, Sos1, TESK, and FRS2 [11–13]. However, a role for Spry2 in enhancing epidermal growth factor (EGF)-induced extracellular signal-regulated kinase 1/2 (ERK1/2) activation by preventing c-Cbl–mediated degradation of EGF receptor has also been described [14]. We have shown a positive role for Spry2 in inducing sustained phosphorylation of ERK1/2 leading to its bistability in airway epithelial cells upon repetitive stimulation [15]. In contrast, overexpression of Spry2 in B cells compromised B cell receptor (BCR)-triggered ERK1/2 activation by engaging and antagonizing Syk, RAF, and BRAF [16]. Similarly, Spry1-deficient T cells [17] or Spry1/2-deficient antigen-specific memory CD8^+^ T cells [18] showed increased proliferation in response to T cell receptor (TCR) stimulation, suggesting a negative regulatory effect. While ectopically expressed Spry1 has a positive effect on TCR signaling in naïve T cells, it has an opposite effect in differentiated Th1 clones [19]. Collectively, these results demonstrate the context-dependent ability of Spry to function as either a positive or a negative regulator of ERK1/2 activation.

ERK1/2 activation is directly implicated in T cell maturation [20], activation [21], and Th2 differentiation [22,23]. TCR ligation triggers the assembly of a multimolecular complex with concomitant phosphorylation of the ζ-chain of the CD3 complex by the kinase LCK [24,25]. This dynamic and complex process links activation of cell surface receptors to nuclear effectors and subsequent transcription of cytokines [25]. Although several molecular mechanisms of ERK1/2 activation have been reported [20,21,23,26], our understanding of ERK1/2 regulatory mechanisms is incomplete. In this study, we for the first time show that Spry2 positively regulated LCK and ERK1/2 activation and CD4^+^ T cell function by modulating the recruitment and activity of CSK.

## Results

### Effect of Spry2 ablation on immune cells

**Fig 1:**
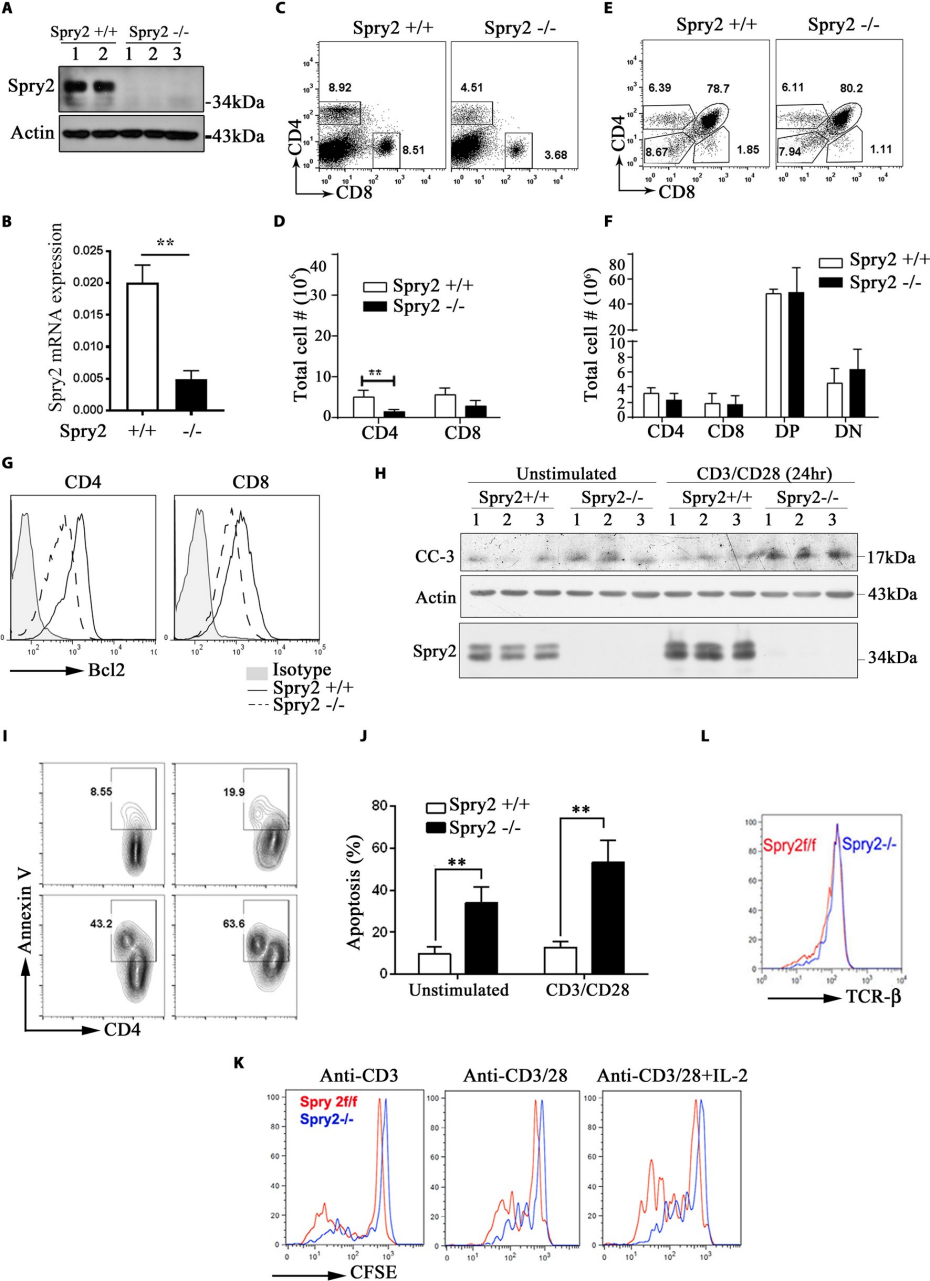
Effect of Spry2 deletion on T cells. **(A)** CD4^+^ T cells from Tamoxifen-treated Spry2^f/f^ (*Spry2*^*+/+*^) (*n =* 2) and ERT^2^-Cre:Spry2^f/f^ (*Spry2*^*−/−*^) mice (*n =* 3) analyzed by western blot to confirm Tamoxifen-inducible deletion of Spry2. **(B)** Real-time PCR analysis of basal Spry2 mRNA expression in splenic CD4^+^ T cells from *Spry2*^*+/+*^ and *Spry2*^*−/−*^ mice (*n =* 3). **(C, D)** Representative dot plot and statistical analysis of splenic CD4^+^ and CD8^+^ T cells from *Spry2*^*+/+*^ and *Spry2*^*−/−*^ mice (*n =* 3). **(E, F)** Representative dot plot and statistical analysis of SP CD4^+^, CD8^+^, DP and DN thymocytes from *Spry2*^*+/+*^ and *Spry2*^*−/−*^ mice (*n =* 3). **(G)** Histogram of Bcl2 expression in splenic CD4^+^ and CD8^+^ T cells from *Spry2*^*+/+*^ and *Spry2*^*−/−*^ mice (*n* = 3). **(H)** Western blot showing CC-3 levels in splenic CD4^+^ T cells from *Spry2*^*+/+*^ and *Spry2*^*−/−*^ mice (*n =* 3). **(I, J)** Annexin-V staining analysis of unstimulated or anti-CD3/CD28-stimulated CD4^+^ T cells from *Spry2*^*+/+*^ and *Spry2*^*−/−*^ mice (*n =* 3). **(K)** Proliferation analysis of CFSE-labeled CD4^+^ T cells from *Spry2*^*+/+*^ and *Spry2*^*−/−*^ mice after 72 h culture (*n* = 3) **(L)** Histogram of surface TCR-β expression in CD4^+^ T cells from *Spry2*^*+/+*^ and *Spry2*^*−/−*^ mice. Values represent mean ± SEM. ** *p* < 0.005 by unpaired Student *t* test. All the data of this figure can be found in the S1 and S2 Data files. Bcl2, B cell lymphoma 2; CC-3, Cleaved Caspase-3; CFSE, carboxyfluorescein succinimidyl ester; DN, double-negative; DP, double-positive; SP, single-positive; Spry2, Sprouty2; TCR-β, T cell receptor beta.

To study the role of Spry2, we generated ERT^2^-Cre:*Spry2*^*flox/flox*^ mice, which allows conditional deletion of Spry2 upon Tamoxifen administration (*Spry2*^*−/−*^). Tamoxifen treatment eliminated Spry2 protein (Fig 1A) and mRNA (Fig 1B) expression in T cells. Tamoxifen-treated *Spry2*^*f/f*^ and ERT^2^-Cre had a normal level of Spry2, and we used Tamoxifen-treated *Spry2*^*f/f*^ as controls (*Spry2*^*+/+*^). Flow cytometric analysis revealed a significantly reduced CD4^+^ but not CD8^+^ T cells in the spleens of *Spry2*^*−/−*^ mice (Fig 1C and 1D). The numbers of double-positive (DP), single-positive (SP) CD4^+^, and SP CD8^+^ were similar in the thymus from *Spry2*^*−/−*^ and *Spry2*^*+/+*^ mice (Fig 1E and 1F), indicating that Spry2 was dispensable for transition from double-negative to DP and then SP T cell selection. To assess if reduced T cell numbers in *Spry2*^*−/−*^ mice was due to Tamoxifen toxicity, we generated mice with CD4-targeted Spry2 deletion by crossing *Spry2*^*f/f*^ mice with CD4-Cre mice (S1A Fig). Similar to ERT^2^-Cre:*Spry2*^*flox/flox*^ mice, CD4-Cre:*Spry2*^*f/f*^ mice had reduced peripheral CD4^+^ T cell numbers compared to *Spry2*^*f/f*^ littermate controls (S1B Fig).

Reduced T cell numbers in peripheral organs could be due to survival defects. Hence, splenic T cells from *Spry2*^*+/+*^ and *Spry2*^*−/−*^ mice were assayed for B cell lymphoma 2 (Bcl-2), a survival protein. Bcl-2 expression in CD4^+^ T cells from *Spry2*^*−/−*^ mice was compromised (Fig 1G), suggesting that Spry2 promotes survival. Cleaved Caspase-3 levels (Fig 1H) and Annexin-V staining (Fig 1I and 1J) in *Spry2*^*−/−*^ CD4^+^ T cells was increased upon anti-CD3/CD28 stimulation, suggesting increased apoptosis. CD4^+^ T cells from *Spry2*^*−/−*^ mice showed defective proliferation as shown by the carboxyfluorescein succinimidyl ester (CFSE) dilution assay, especially in response to anti-CD3/CD28+IL2 stimulation (Fig 1K, inducible knockout; S1C Fig, CD4-targeted knockout). The expression of TCRβ in *Spry2*^*−/−*^ was comparable to *Spry2*^*+/+*^ mice (Fig 1L). Collectively, these results suggest that Spry2 is critical for survival and proliferation of CD4^+^ T lymphocytes.

### Spry2 deficiency abrogates ERK1/2 activation, T cell differentiation, and cytokine secretion

**Fig 2:**
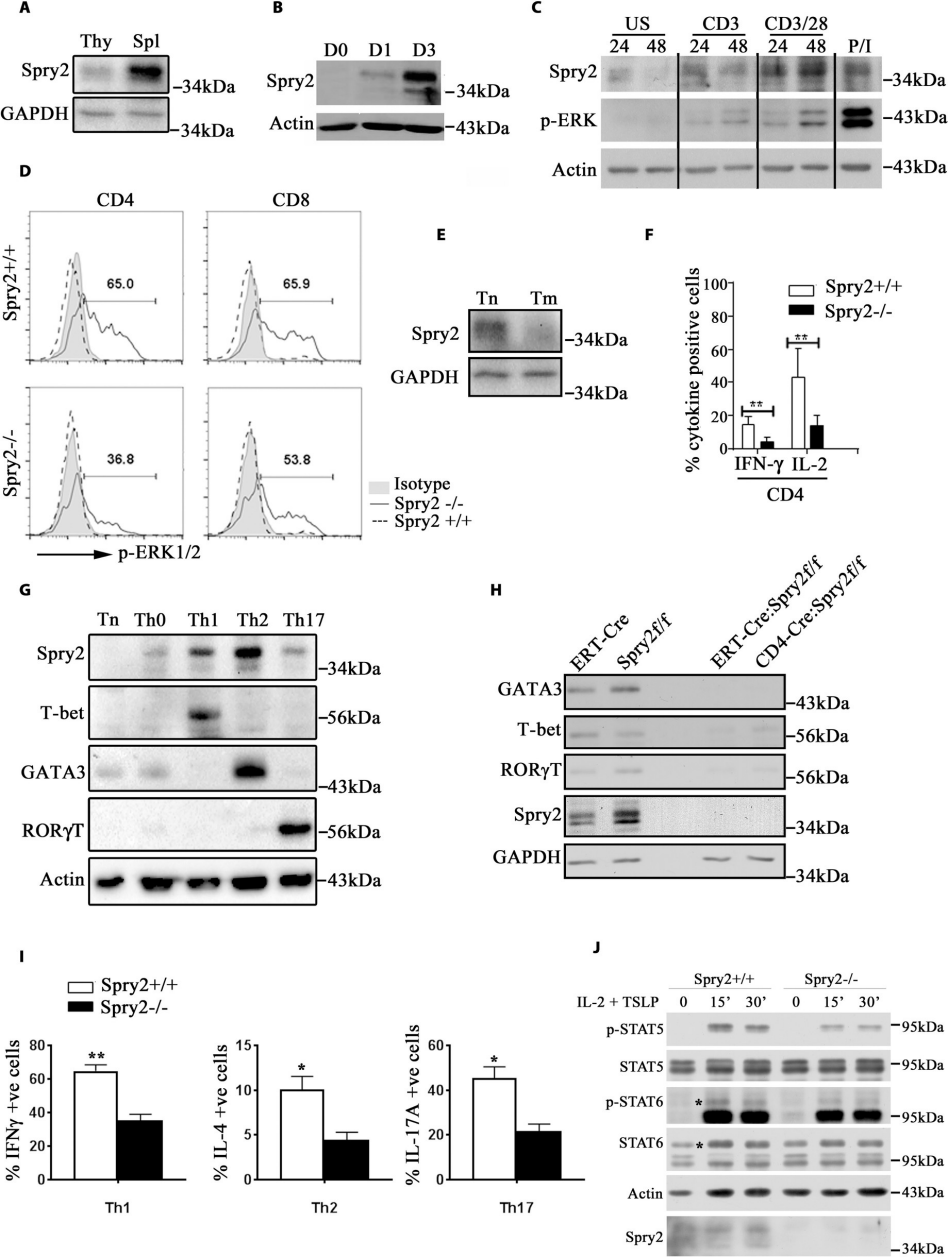
Spry2 deficiency abrogates ERK activation, T cell differentiation, and cytokine secretion. **(A)** Immunoblot of basal Spry2 expression in Thy and Spl from B6 mice (*n =* 2) **(B)** and Spry2 protein induction in anti-CD3/CD28-stimulated CD4^+^ T cells (Day 0, 1, and 3) (*n =* 3). **(C)** Immunoblot of Spry2 protein induction and ERK activation in unstimulated and stimulated human CD4^+^ T cells at indicated time points (24 h and 48 h, respectively); P/I represents phorbol myristate acetate (20 ng/mL) and ionomycin (500 ng/mL) (*n =* 3). **(D)** Histogram of p-ERK1/2 levels in CD4^+^ and CD8^+^ T cells from *Spry2*^*+/+*^ and *Spry2*^*−/−*^ mice stimulated with anti-CD3/CD28 for 24 h. **(E)** Immunoblot of Spry2 expression in Tn and Tm from B6 mice (*n =* 2). **(F)** Graph showing statistical analysis of intracellular IL-2 and IFN-γ in anti-CD3/28-stimulated (24 h) CD4^+^ T cells from *Spry2*^*+/+*^ and *Spry2*^*−/−*^ mice (*n =* 3). **(G)** Immunoblot of Spry2 and transcription factor expression in naïve and differentiated CD4^+^ T cells from C57BL/6 mice (*n =* 3). **(H)** Immunoblot of T-bet, GATA3, and RORγT in CD4^+^ T cells from *Spry2*^*+/+*^ and *Spry2*^*−/−*^ mice (*n* = 2). **(I)** Graph represents percent intracellular cytokine-positive CD4^+^ T cells skewed under Th1, Th2, and Th17 conditions from *Spry2*^*+/+*^ and *Spry2*^*−/−*^ mice (*n* = 3). **(J)** Immunoblot of p-STAT5 and p-STAT6 in CD4^+^ T cells, treated with IL-2 (10 ng/mL) and TSLP (10 ng/mL) at indicated time points (in min). * in the blot indicates the location of p-STAT6/STAT6 bands. Values are mean ± SEM, significance * *p* < 0.05; ** *p* < 0.005 by Student *t* test (*n =* 3). All the data of this figure can be found in the S1 and S2 Data files. ERK, extracellular signal-regulated kinase; IL-2, interleukin 2; IFN-γ, interferon gamma; Spl, splenocyte; Spry2, Sprouty2; Thy, thymocyte; Tm, memory CD4^+^ T cell; Tn, naïve CD4^+^ T cell; TSLP, thymic stromal lymphopoietin.

The differential effect of Spry2 deletion on splenic and thymic T cell populations prompted us to check Spry2 expression in the splenocyte and thymocyte compartments under homeostatic conditions. Spry2 expression was higher in splenocytes as compared to thymocytes in B6 mice (Fig 2A). Further, Spry2 was induced in murine CD4^+^ T cells upon TCR stimulation in a time-dependent manner (Fig 2B). To confirm the inducible nature of Spry2, human CD4^+^ T cells were stimulated and immunoblotted for Spry2 and p-ERK. We observed progressive induction of Spry2 and a positive correlation between Spry2 induction and ERK1/2 activation in a time- and stimulatory strength-dependent manner (Fig 2C). This positive correlation was established in mouse CD4^+^ T cells wherein Spry2 deficiency substantially abrogated ERK1/2 activation in CD4^+^ T cells and modestly in CD8^+^ T cells (Fig 2D). Further, under homeostatic conditions, Spry2 expression was higher in splenic naïve CD4^+^ T cells relative to memory CD4^+^ T cells (Fig 2E).

Activated CD4^+^ T cells from *Spry2*^*−/−*^ mice expressed significantly less IL-2 and interferon gamma (IFN-γ) (Fig 2F). To test the role of Spry2 in T helper cell differentiation, naïve CD4^+^ T cells (Tn) cultured in the absence (Th0) or presence of Th1-, Th2-, or Th17-polarizing cytokines. We observed higher Spry2 expression under Th1-, and especially under Th2- and, minimally, under Th17-skewing conditions (Fig 2G). The expression of T-bet, GATA-3, and RORγT was compromised under homeostatic conditions in both ERT^2^-Cre:*Spry2*^*flox/flox*^ and CD4-Cre:*Spry2*^*flox/flox*^ mice (Fig 2H). Next, CD4^+^ T cells from *Spry2*^*+/+*^ and *Spry2*^*−/−*^ mice were polarized under Th1, Th2, and Th17 conditions and evaluated for expression of IFN-γ, IL-4, and IL-17, respectively (Figs 2I and S2A). IFN-γ^+^, IL-4^+^, and IL-17^+^ CD4^+^ T cells were decreased in *Spry2*^*−/−*^ mice. Differentiation of IFN-γ^+^ Th1 and IL-4^+^ Th2 cells in CD4-Cre:*Spry2*^*f/f*^ mice was similarly reduced (S1E Fig). STAT signaling plays a critical role in T helper cell differentiation. Since T helper cell differentiation was inhibited in *Spry2*^*−/−*^ mice, we examined STAT5 and STAT6 (activating) phosphorylation. Stimulation of spleen CD4^+^ T cells with IL2 plus thymic stromal lymphopoietin (TSLP) induced phosphorylation of STAT5 and, to a lower extent, STAT6. This was attenuated in *Spry2*^*−/−*^ mice (Fig 2J). In addition, under homeostatic conditions, the surface expression of IL2 receptor (CD25) is reduced in *Spry2*^*−/−*^ CD4^+^ T cells relative to *Spry2*^*+/+*^ CD4^+^ T cells (S2B Fig). These data suggest that Spry2 is necessary for T helper cell differentiation and cytokine production.

### CD4^+^ T cell Spry2 drives asthma

**Fig 3:**
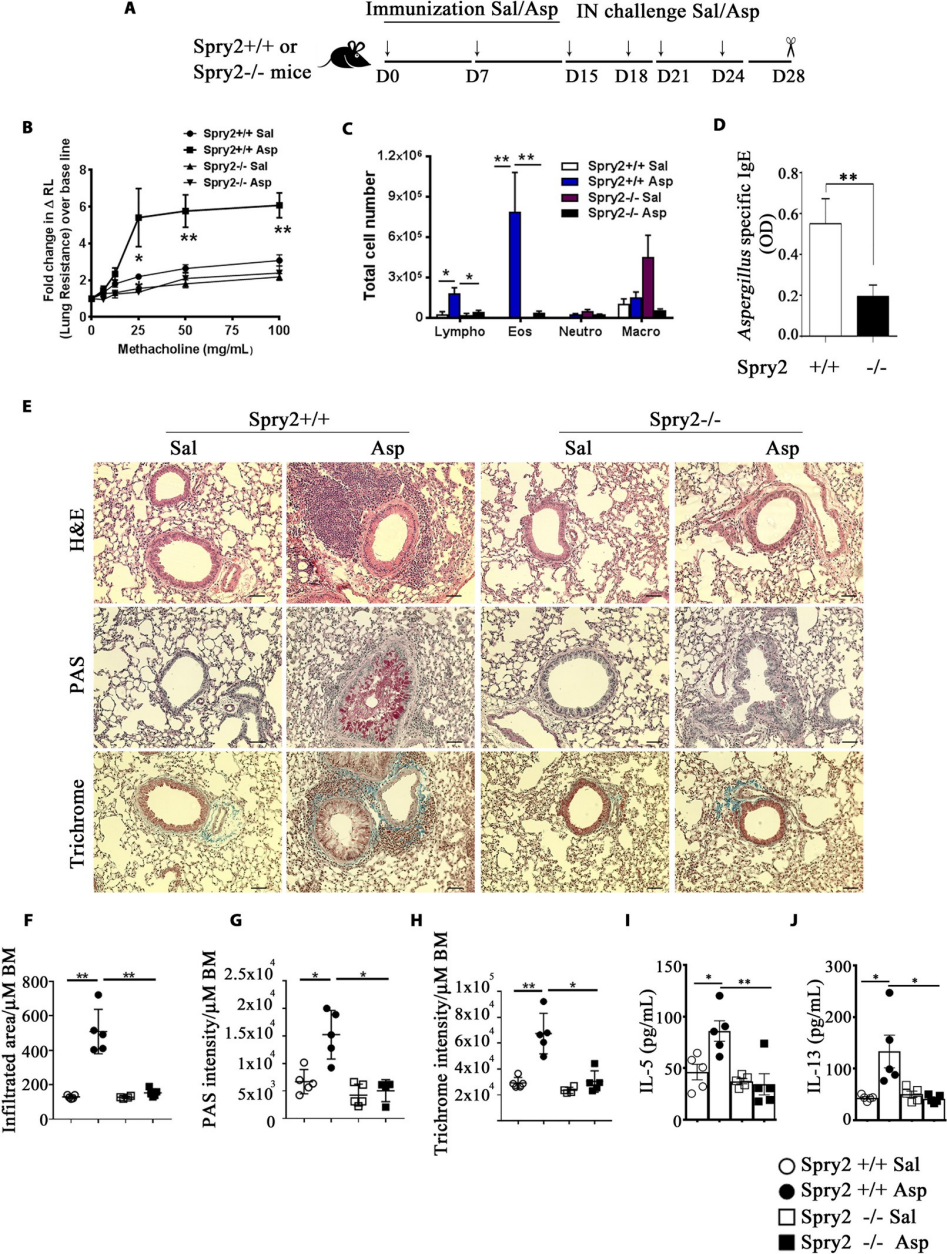
CD4^+^ T cell Spry2 drives asthma. **(A)** A schematic diagram of a Th2-driven asthma model for immunization with *Asp* or Sal, IN challenge, and analysis. **(B)** AHR (change in lung resistance over baseline) to inhaled methacholine as measured by flexiVent. **(C)** Total number of Lympho, Eos, Macro, and Neutro in BAL fluid (*n =* 5/group). **(D)** Serum *Asp*-specific IgE antibody in *Spry2*^*+/+*^ and *Spry2*^*−/−*^ mice sensitized and challenged with *Asp* as in **(A)** (*n* = 3). **(E)** Representative H&E staining (inflammation), PAS staining (mucus), and Trichrome staining (collagen deposition) from lungs of *Spry2*^*+/+*^ and *Spry2*^*−/−*^ mice sensitized and challenged with Sal or *Asp* as indicated in **Fig 3A.** Scale bar, 100 μm. Lung morphometric analysis of peribronchial and perivascular inflamed area **(F)**, epithelial PAS staining intensity **(G)**, and peribronchial and perivascular Trichrome staining intensity **(H)** per micrometer (μm) of bronchial BM. *Spry2*^*+/+*^ and *Spry2*^*−/−*^ mice sensitized and challenged with Sal or *Asp* as in **(A),** IL-5 **(I),** and IL-13 **(J)** in BALF were analyzed by ELISA. Data are represented as mean +/− SEM. Significance * *p* < 0.05; ** *p* < 0.005 by Student *t* test. (*n =* 5 mice/group). All the data of this figure can be found in the S1 Data file. AHR, airway hyperresponsiveness; *Asp*, *Aspergillus*; BAL, bronchoalveolar lavage; BALF, bronchoalveolar lavage fluid; BM, basement membrane; Eos, eosinophils; H&E, hematoxylin and eosin; IgE, immunoglobulin E; IN, intranasal; Lympho, lymphocyte; Macro, macrophage; Neutro, neutrophil; PAS, periodic acid–Schiff; Sal, saline; Spry2, Sprouty2.

Next, we studied the role of Spry2 in airway type 2 inflammation in a mouse model of allergic asthma. *Spry2*^*+/+*^ and *Spry2*^*−/−*^ mice were sensitized and intranasally (IN) challenged with saline or *Aspergillus* (*Asp*) extract (Fig 3A) and assessed for airway resistance, inflammatory cell influx into the airways, and lung inflammation [27]. Airway hyperresponsiveness (AHR) to methacholine and bronchoalveolar lavage (BAL) eosinophils and lymphocytes were decreased in *Spry2*^*−/−*^ mice (Fig 3B and 3C). *Asp*-specific IgE was significantly lower in the serum of *Asp*-treated *Spry2*^*−/−*^ mice (Fig 3D). Likewise, lung inflammatory cell infiltrates, mucous production, and collagen deposition were substantially reduced (Fig 3E–3H). Further, IN administration of *Asp* extract in *Spry2*^*+/+*^ mice resulted in significant accumulation of type 2 cytokines IL-5 and IL-13 in the bronchoalveolar lavage fluid (BALF) as compared to saline administered counterparts. However, these cytokine levels were significantly decreased in the BALF of *Asp*-treated *Spry2*^*−/−*^ mice (Fig 3I and 3J). Ovalbumin-induced asthma in CD4-Cre:*Spry2*^*f/f*^ mice had similarly reduced lung inflammation (S1F and S1G Fig). These results suggest that Spry2 contributes to airway eosinophilic inflammation, AHR, mucous production, and collagen deposition upon *Asp* exposure.

**Fig 4:**
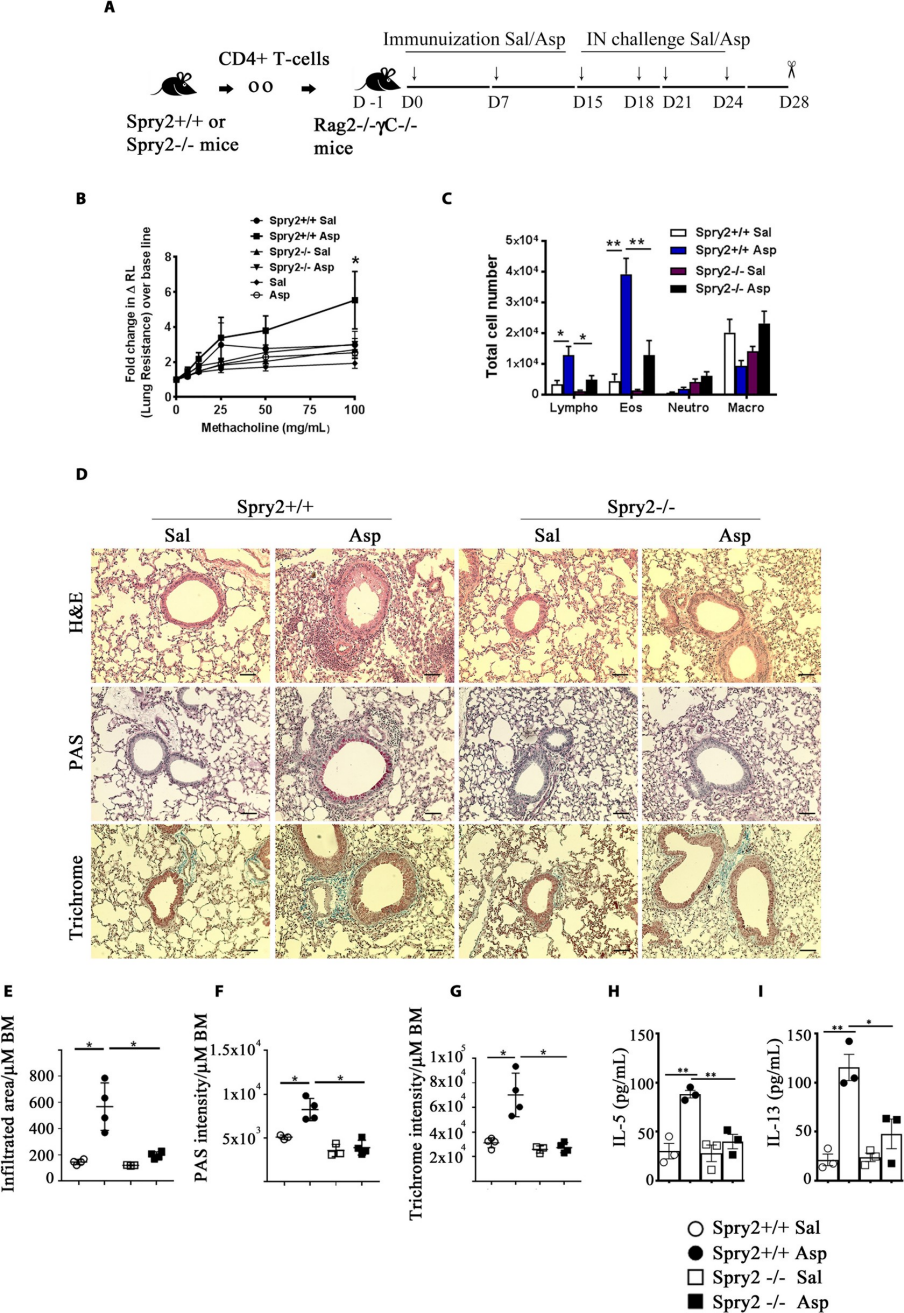
Adoptive transfer of *Spry2*^*−/−*^ CD4^+^ T cells prevents *Aspergillus*-induced asthma in *Rag2*^*−/-*^*γC*^*−/−*^ mice. **(A)** Diagram of asthma model for adoptive transfer, immunization, IN challenge, and analysis. **(B)** AHR to inhaled methacholine as measured by flexiVent. *Spry2*^*+/+*^ Sal and *Spry2*^*+/+*^ Asp represents groups in which CD4^+^ T cells from *Spry2*^*+/+*^ mice were adoptively transferred to *Rag2*^*−/-*^*γC*^*−/−*^ mice and subsequently challenged with Sal or *Asp* as indicated in **(A)**. *Spry2*^*−/−*^ Sal and *Spry2*^*−/−*^ Asp represents groups in which CD4^+^ T cells from *Spry2*^*−/−*^ mice were adoptively transferred to *Rag2*^*−/-*^*γC*^*−/−*^ mice and challenged with Sal or *Asp* as indicated in **(A)**. Sal and Asp groups in **(B)** indicates naïve *Rag2*^*−/-*^*γC*^*−/−*^ mice challenged with Sal or *Asp*. **(C)** Graph showing total number of Lympho, Macro, Eos, and Neutro in BAL fluid. **(D**) Representative H&E, PAS, and Trichrome staining from lungs of recipient *Rag2*^*−/-*^*γC*^*−/−*^ mice. Scale bar, 100 μm. Lung morphometric analysis of peribronchial and perivascular inflamed area **(E)**, epithelial PAS staining intensity **(F)**, and peribronchial and perivascular trichrome staining intensity **(G)** per μm of BM. Recipient *Rag2*^*−/-*^*γC*^*−/−*^ mice were IN challenged as indicated in **(A);** IL-5 **(H)** and IL-13 **(I)** in BALF were analyzed by ELISA. Data are represented as mean +/− SEM. Significance * *p* < 0.05; ** *p* < 0.005 by Student *t* test. (*n =* 3 to 4 mice/group). All the data of this figure can be found in the S1 Data file. AHR, airway hyperresponsiveness; *Asp*, *Aspergillus*; BAL, bronchoalveolar lavage; BALF, bronchoalveolar lavage fluid; BM, basement membrane; Eos, eosinophils; H&E, hematoxylin and eosin; IN, intranasal; Lympho, lymphocyte; Macro, macrophage; Neutro, neutrophil; PAS, periodic acid–Schiff; Sal, saline.

To assess the role of Th2 cells independent of other type-2 innate immune cells, we adoptively transferred CD4^+^ T cells from *Spry2*^*+/+*^ and *Spry2*^*−/−*^ mice to *Rag2*^*−/-*^*γC*^*−/−*^ mice lacking ILC2s. Recipient mice were sensitized and IN challenged either with Sal or *Asp* (Fig 4A) and assessed for AHR, BAL inflammatory cell influx, and lung inflammation. Consistent with our previous results, mice receiving *Spry2*^*−/−*^ CD4^+^ T cells showed reduced AHR, decreased eosinophils and lymphocytes in the BAL, attenuated lung inflammation, mucus production, and collagen deposition upon *Asp* exposure (Fig 4B–4G). Similarly, secretion of IL5 and IL13 was severely impaired in allergen-challenged *Rag2*^*−/-*^*γC*^*−/−*^ mice that received CD4^+^ T cells from *Spry2*^*−/−*^ mice, as compared to the recipient mice which received CD4^+^ T cells from *Spry2*^*+/+*^ mice (Fig 4H and 4I). These data suggest that Spry2 positively regulates T cell–driven asthma by up-regulating Th2 cytokines.

### Spry2 expression is increased in asthma

**Fig 5:**
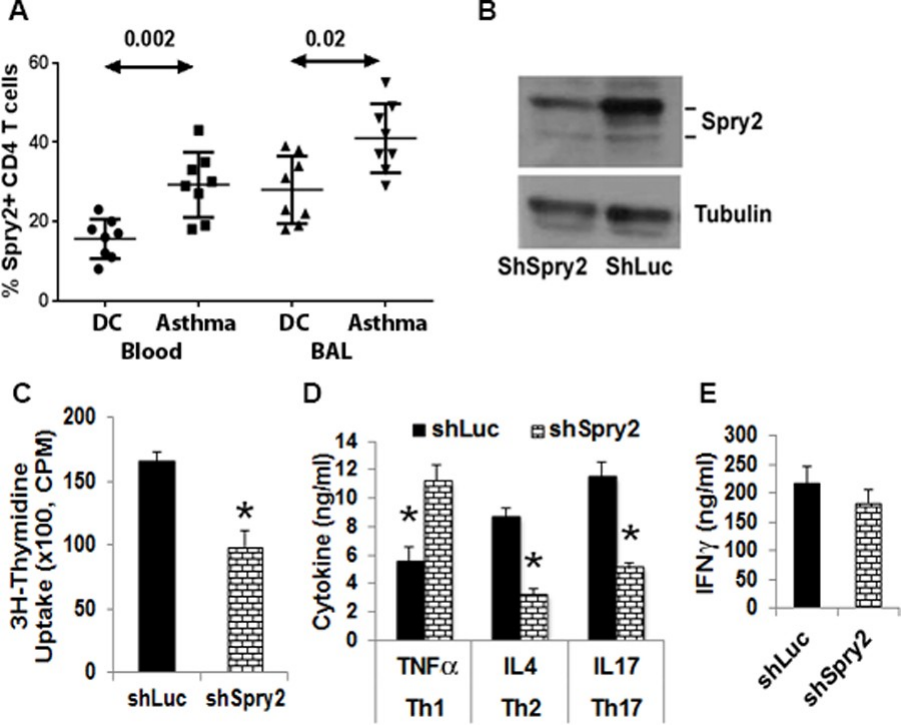
Expression of Spry2 in human CD4^+^ T cells. **(A)** PBMCs and BAL fluid from patients with allergic asthma or DC were stained for Spry2 and CD4, then analyzed by flow cytometry. Frequency of Spry2^+^ CD4^+^ T cells was analyzed by Mann–Whitney *U* test (*n =* 8 donors/group). **(B–E)** Effect of shRNA-mediated knockdown on Spry2 expression **(B)**, CD4^+^ T cell proliferation **(C)**, and cytokine production under Th1- **(D, E)**, Th2- **(D)**, or Th17- **(D)** skewing conditions. Significance * *p* < 0.05, paired *t* test, (*n* = 4/group). All the data of this figure can be found in the S1 and S2 Data files. BAL, bronchoalveolar lavage; CPM, counts per minute; DC, disease control; IFN-γ, interferon gamma; PBMC, peripheral blood mononuclear cell; shLuc, luciferase shRNA; shRNA, short hairpin RNA; shSpry2, Spry2 shRNA; Spry2, Sprouty2.

To further evaluate its contribution to human asthma, we assayed Spry2 expression in CD4^+^ T cells from blood and BAL from patients with asthma and disease controls by flow cytometry. Demographic and clinical characteristics of patients with asthma and disease control are in S1 Table. The expression level of Spry2 in CD4^+^ T cells from blood and BAL was significantly higher in patients with asthma (Fig 5A).

### Spry2 is important for human CD4^+^ T cell function

To investigate the role of Spry2 in human lymphocyte function, we knocked down mRNA expression via short hairpin RNA (shRNA) in a green fluorescent protein (GFP)-expressing lentivirus (Fig 5B). GFP-expressing cells were sorted and examined for function. Lentiviral transduction of Spry2 ShRNA (ShSPRY2), but not control ShRNA (ShLuc), resulted in reduced proliferation of CD4^+^ T cells (measured by thymidine uptake) in response to anti-CD3/CD28 stimulation (Fig 5C). Next, the effect of Spry2 deficiency on T helper cell cytokine production under polarizing conditions was analyzed by ELISA. Spry2 knockdown augmented tumor necrosis factor alpha (TNF-α) with no effect on IFN-γ under Th1-skewing conditions (Fig 5D and 5E). Spry2 deficiency resulted in reduced IL-4 and IL-17 under Th2- and Th17-skewing conditions, respectively (Fig 5D).

### Spry2 deletion results in defective TCR signaling

**Fig 6:**
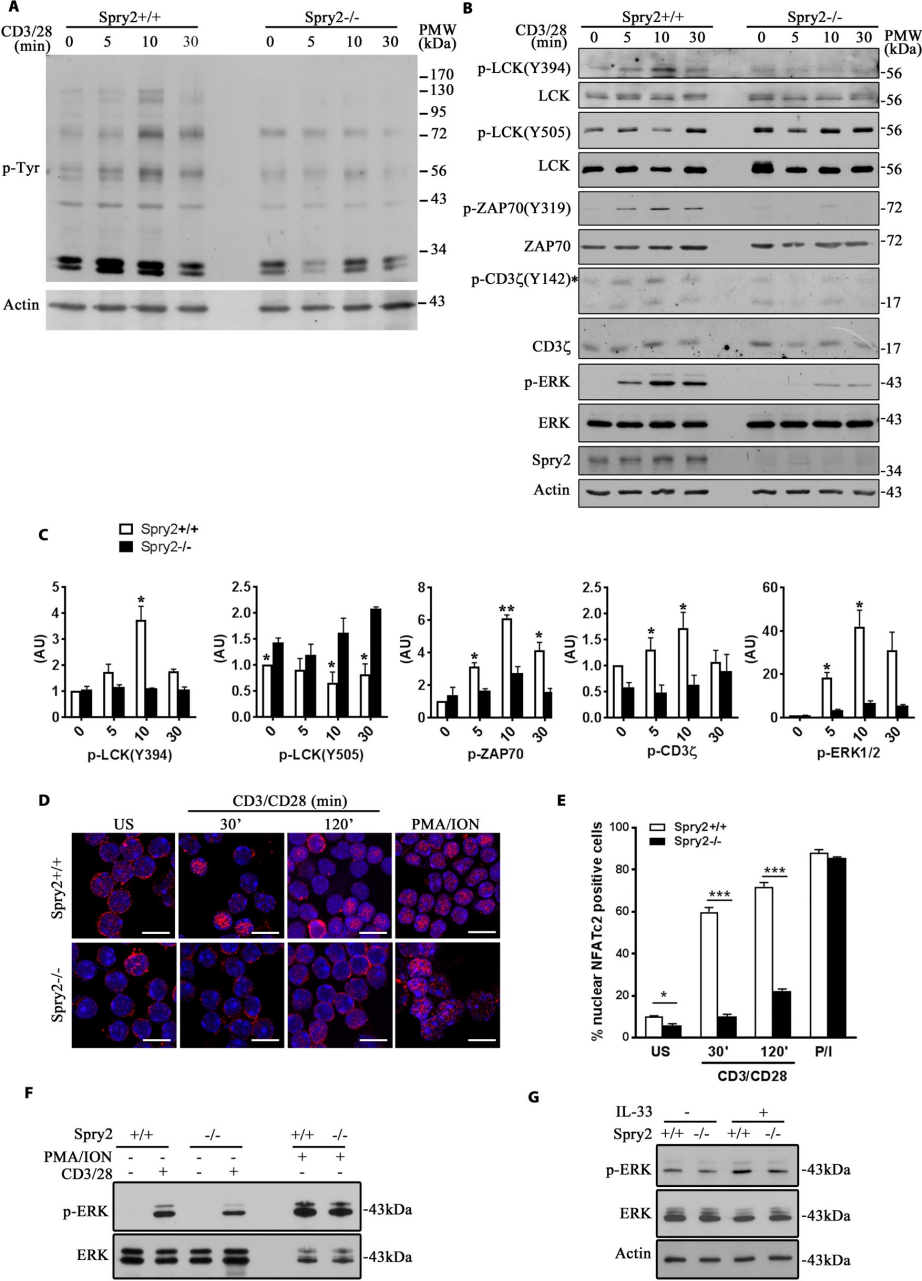
Defective TCR signaling in *Spry2*^*−/−*^ mice. **(A)** p-Tyr and β-Actin immunoblots of sort-purified, anti-CD3/CD28-stimulated CD4^+^ T cells from *Spry2*^*+/+*^ and *Spry2*^*−/−*^ mice (*n =* 3). **(B)** LCK, ZAP70, CD3ζ, and ERK immunoblots of sort-purified, anti-CD3/28-stimulated CD4^+^ T cells from *Spry2*^*+/+*^ and *Spry2*^*−/−*^ mice. * indicates the location of p-CD3ζ bands. (**C**) Quantification of phosphorylated proteins downstream of TCR from *Spry2*^*+/+*^ and *Spry2*^*−/−*^ CD4^+^ T cells stimulated for various time points as indicated in (B). The level of protein phosphorylation is normalized to the respective total protein level at 0 min time point in *Spry2*^*+/+*^ CD4^+^ T cells. Graphs indicate the density ratio (AU) of the phosphorylated protein to the respective total protein at each time point (*n =* 3). **(D, E)** Immunofluorescence analysis of NFATc2 nuclear translocation and quantification in US or anti-CD3/CD28- or PMA/ionomycin-stimulated CD4^+^ T cells from *Spry2*^*+/+*^ and *Spry2*^*−/−*^ mice (*n =* 3). **(F, G)** p-ERK1/2 and ERK1/2 immunoblots of CD4^+^ T cells from *Spry2*^*+/+*^ and *Spry2*^*−/−*^ mice stimulated with anti-CD3/CD28 or PMA/ionomycin for 10 min **(F)** or recombinant IL-33 (20 ng/mL) for 30 min **(G)** (*n* = 3). Significance * *p* < 0.05; *** *p* < 0.0001 by Student *t* test. All the data of this figure can be found in the S1 and S2 Data files. β-Actin, beta actin; AU, arbitrary unit; ERK, extracellular signal-regulated kinase; PMA, phorbol myristate acetate; p-Tyr, phosphotyrosine; Spry2, Sprouty2; TCR, T cell receptor; US, unstimulated.

TCR ligation activates LCK [25] culminating in activation of ERK1/2, NFAT, and NFκB signaling modules [24,25,28]. We investigated the effect of Spry2 on TCR signaling. Temporal analysis of global phospho-tyrosine levels upon TCR stimulation revealed significant inhibition of tyrosine phosphorylation in *Spry2*^*−/−*^ CD4^+^ T cells (Fig 6A). *Spry2*^*+/+*^ CD4^+^ T cells manifested strong activating phosphorylation of LCK (Y394) upon TCR stimulation (Fig 6B and 6C). In contrast, *Spry2*^*−/−*^ CD4^+^ T cells failed to phosphorylate LCK-Y394. The phosphorylation of the inhibitory tyrosine residue Y505 of LCK was higher in *Spry2*^*−/−*^ CD4^+^ T cells under basal conditions, which further increased after 10 min of TCR ligation (Fig 6B and 6C). Reduced pY394 and elevated pY505 suggested that LCK activation was impaired, which is reflected in defective downstream phosphorylation of ZAP70, CD3ζ, and ERK1/2 in *Spry2*^*−/−*^ CD4^+^ T cells (Fig 6B and 6C). This defective ERK1/2 phosphorylation was confirmed in T cells obtained from CD4-Cre:*Spry2*^*f/f*^ mice (S1D Fig). *Spry2*^*−/−*^ CD4^+^ T cells showed decreased nuclear translocation of NFATc2 as compared to *Spry2*^*+/+*^CD4^+^ T cells under stimulatory conditions (Fig 6D and 6E). This defective NFATc2 nuclear translocation was TCR dependent, as stimulation with phorbol myristate acetate (PMA)/ionomycin resulted in activation of NFATc2 similar to that observed in *Spry2*^*+/+*^ CD4^+^ T cells. To interrogate whether reduced ERK1/2 phosphorylation in *Spry2*^*−/−*^ T cells was TCR dependent, we stimulated *Spry2*^*+/+*^ or *Spry2*^*−/−*^ CD4^+^ T cells either with anti-CD3/CD28, PMA/ionomycin, or recombinant IL-33. The PMA/ionomycin and IL-33–stimulated ERK1/2 phosphorylation in both groups was similar (Fig 6F and 6G), suggesting that reduced ERK1/2 phosphorylation in *Spry2*^*−/−*^ CD4^+^ T cells was TCR dependent. Together, these results demonstrate that Spry2 affects TCR-mediated Y394 phosphorylation of LCK, activating phosphorylation of ERK1/2 and nuclear translocation of NFATc2.

### Enhanced interaction between CSK and LCK in *Spry2*^−/−^ CD4^+^ T cells

**Fig 7:**
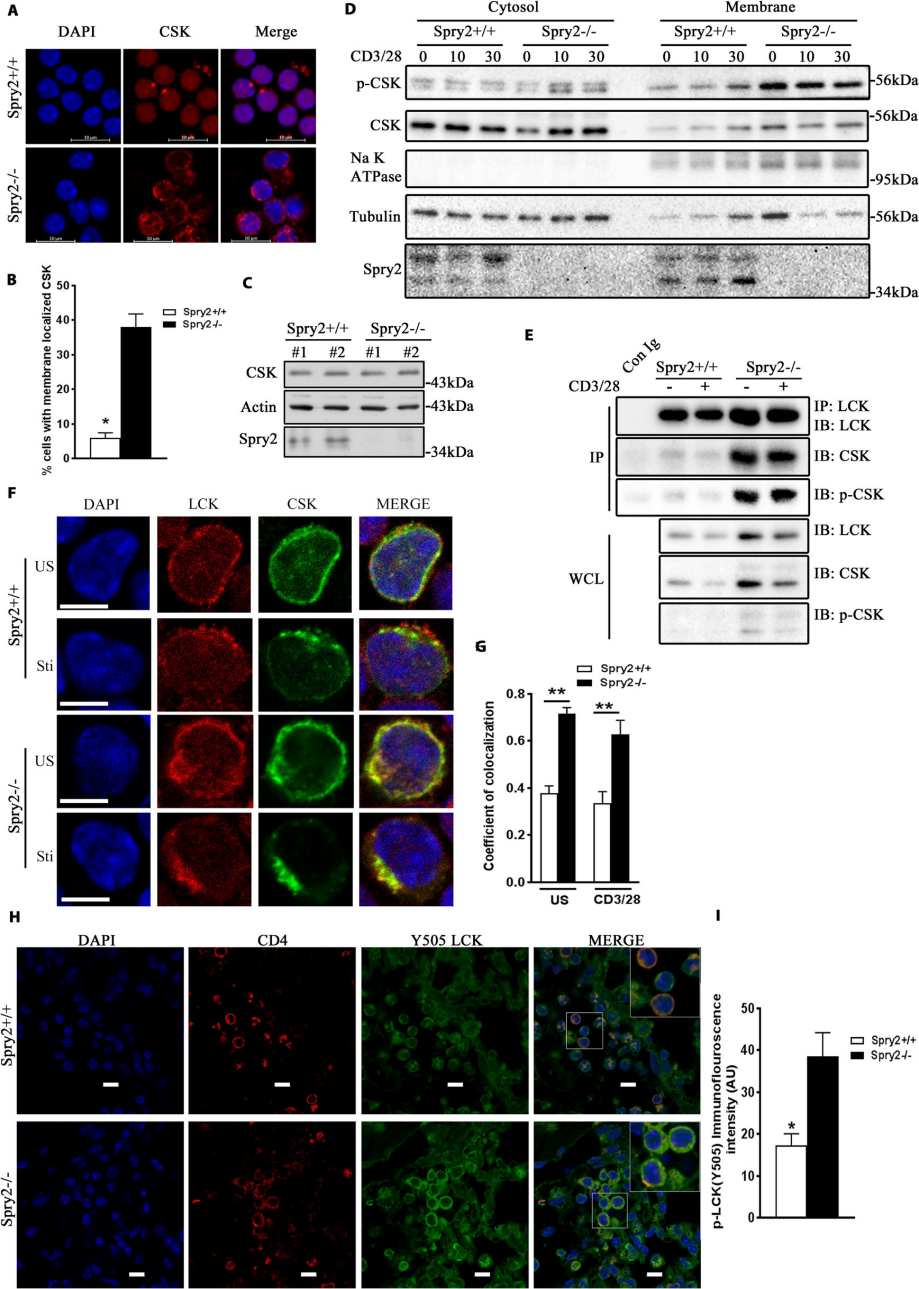
Enhanced interaction between CSK and LCK in *Spry2*^*−/−*^ CD4^+^ T cells. **(A, B)** Representative confocal images showing localization of CSK **(A)** and enumeration of membrane localized CSK **(B)** in CD4^+^ T cells from *Spry2*^*+/+*^ and *Spry2*^*−/−*^ mice (*n =* 3). **(C)** Immunoblot analysis of total CSK levels in *Spry2*^*+/+*^ and *Spry2*^*−/−*^ CD4^+^ T cells (*n =* 2). **(D)** p-CSK and CSK immunoblots of cytosol and membrane fractions of stimulated CD4^+^ T cells from *Spry2*^*+/+*^ and *Spry2*^*−/−*^ mice. Na^+^ K^+^ ATPase and Tubulin serve as loading controls for membrane and cytosolic fractions, respectively (*n =* 3). **(E)** Immunoprecipitation of LCK and immunoblotting of LCK, CSK, and p-CSK in CD4^+^ T cells from *Spry2*^*+/+*^ and *Spry2*^*−/−*^ mice under unstimulated and anti-CD3/CD28-stimulated (10 min) conditions (*n =* 3). **(F)** Representative confocal images of LCK- and CSK-stained CD4^+^ T cells from *Spry2*^*+/+*^ and *Spry2*^*−/−*^ mice under US and anti-CD3/CD28-stimulated (10 min) conditions. (*n* = 3) Scale bar, 5 μm. **(G)** A graphical presentation of Pearson correlation coefficient of colocalization of CSK with LCK, *n* = 25 cells per experimental group. **(H)** Representative confocal images of CD4 and p-LCK (Y505) immunofluorescence staining of lung sections from *Asp*-challenged *Spry2*^*+/+*^ and *Spry2*^*−/−*^ mice from the asthma model. Scale bar, 10 μm **(I)** Statistical analysis of the CTCF intensity [represented as AU of p-LCK(Y505)] from individual CD4^+^ T cells shown in **(H)**. Significance * *p* < 0.05, ** *p* < 0.005, by Student *t* test. (*n =* 3). All the data of this figure can be found in the S1 and S2 Data files. *Asp*, *Aspergillus*; AU, arbitrary unit; CTCF, corrected total cellular fluorescence; IB, immunoblotting; IP, immunoprecipitation; Spry2, Sprouty2; Sti, stimulated; US, unstimulated; WCL,whole cell lysate.

LCK is negatively regulated by CSK. The latter is predominantly cytosolic and requires membrane adapters in the vicinity of LCK-containing lipid rafts to exert its inhibitory effect [29]. In addition, phosphorylation of CSK at Ser-364 results in increased kinase activity [30]. Thus, the membrane-bound active CSK phosphorylates LCK at Y505 to suppress TCR signaling. We determined CSK subcellular localization in CD4^+^ T cells. CSK was diffusely distributed in the cytosol of *Spry2*^*+/+*^ CD4^+^ T cells, whereas a major fraction of CSK was localized to the plasma membrane in *Spry2*^*−/−*^ CD4^+^ T cells (Fig 7A and 7B). However, the total levels of CSK remained the same in *Spry2*^*+/+*^ and *Spry2*^*−/−*^ CD4^+^ T cells (Fig 7C), suggesting that it is the altered membrane localization of CSK that is responsible for increased LCK(Y505) phosphorylation in *Spry2*^*−/−*^ CD4^+^ T cells. Subcellular fractionation and immunoblotting showed increased levels of p-CSK and CSK in the membrane fractions of *Spry2*^*−/−*^ CD4^+^ T cells (Fig 7D). Immunoprecipitation studies showed that higher levels of CSK coprecipitated with LCK from *Spry2*^*−/−*^ CD4^+^ T cells and that enhanced LCK:CSK interaction persisted for 10 min after anti-CD3/CD28 stimulation (Fig 7E). We examined the phosphorylation status of CSK in the immunoprecipitates and found higher level of p-CSK in *Spry2*^*−/−*^ CD4^+^ T cells relative to controls (Fig 7E). Immunostaining of LCK and CSK under unstimulated and stimulated conditions (Fig 7F and 7G) showed enhanced colocalization of LCK and CSK in *Spry2*^*−/−*^ CD4^+^ T cells. Enhanced colocalization with CSK may increase phosphorylation (Y505) of LCK. We therefore examined in situ LCK(Y505) phosphorylation in CD4^+^ T cells from the lung sections obtained from our mouse asthma model and observed increased phospho-LCK (Y505) staining in CD4^+^ T cells from *Asp*-challenged *Spry2*^*−/−*^ mice (Fig 7H and 7I). Thus, Spry2 deletion alters the localization and activity of CSK, enhanced its interaction with LCK, and augmented LCK Y505 phosphorylation.

### Knockdown of CSK restores ERK1/2 phosphorylation, proliferation, and effector cytokine production in *Spry2*^*−/−*^ CD4^+^ T cells

Our data suggest hyperactive CSK suppresses TCR-driven LCK and ERK1/2 activity in *Spry2*^*−/−*^ CD4^+^ T cells. Therefore, we asked if CSK knockdown would restore TCR-driven ERK1/2 activity, proliferation, and cytokine production in *Spry2*^*−/−*^ CD4^+^ T cells. Lentiviral transduction of CSK ShRNA1 (CSKSh1) and ShRNA3 (CSKSh3) in *Spry2*^*+/+*^ CD4^+^ T cells showed approximately 70% reduction in CSK levels relative to control ShRNA (ConSh)-transduced cells (S3A Fig). Transduction of ConSh had no effect on ERK1/2 phosphorylation in activated *Spry2*^*−/−*^ T cells. However, transduction of CSK Sh1 and Sh3 restored phosphorylation of ERK1/2 (S3B Fig). We then assayed the effect of CSK knockdown on T cell proliferation and cytokine production. In contrast, CSK knockdown reversed the defect in cell proliferation (Ki67 staining) and IFN-γ and IL-4 production in *Spry2*^*−/−*^ CD4^+^ T cells compared to controls (S3C–S3E Fig). Taken together, these results suggest Spry2 regulation of CSK plays a key role in ERK1/2 signaling, T cell proliferation, and cytokine production.

### Enhanced interaction between CSK and Cav-1 leads to increased CSK activity in *Spry2*^*−/−*^ CD4^+^ T cells

Differences in CSK activity prompted us to determine the role of Spry2 in the regulation of CSK. To test whether Spry2 is targeted to the lipid rafts, anti-CD3/CD28-stimulated CD4^+^ T cells were stained for Spry2, CSK, and Cholera Toxin-B (CTX-B, a lipid raft marker). Colocalization of Spry2 with CTX-B was minimal prior to TCR triggering (S4A Fig); however, 5 min post-TCR triggering, Spry2 was associated with CSK in the lipid rafts, which completely diminished 30 min post-TCR stimulation. Hence, we hypothesized that Spry2 regulated LCK activity directly by interfering with the complex formation between LCK and CSK. To test this in a reductionist model, we pulled down LCK and subjected the immunoprecipitates to a kinase reaction in the presence or absence of recombinant CSK (r-CSK) and Spry2 (r-Spry2) (S4B Fig). Addition of r-CSK to the immunoprecipitates enhanced LCK-Y505 phosphorylation (S4B Fig, lane 4). However, r-CSK in the presence of increasing concentrations of r-Spry2 (S4B Fig, lanes 5, 6, and 7) failed to block the kinase activity of recombinant CSK, thereby suggesting that Spry2 may function by altering CSK recruitment but not its inherent activity.

**Fig 8:**
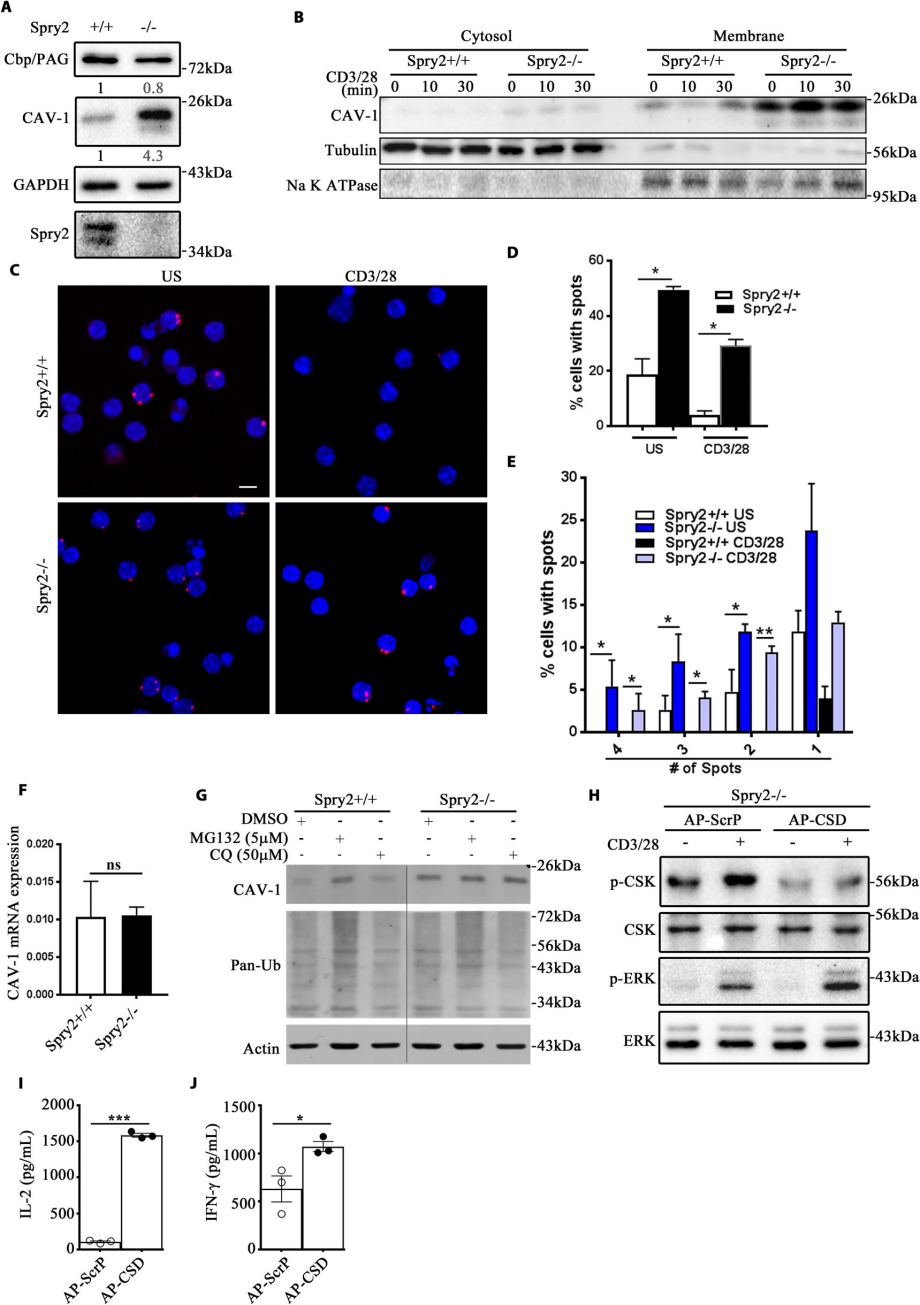
Enhanced CSK:Cav-1 interaction leads to increased CSK activity in *Spry2*^*−/−*^ CD4^+^ T cells. **(A)** Immunoblot of basal Cbp/PAG-1 and Cav-1 levels in CD4^+^ T cells from *Spry2*^*+/+*^ and *Spry2*^*−/−*^ mice (*n =* 3). Numerical values indicate relative densitometric quantification of Cbp/PAG-1 and Cav-1 normalized to GAPDH. **(B)** Immunoblots of Cav-1 from cytosol and membrane fractions of stimulated CD4^+^ T cells from *Spry2*^*+/+*^ and *Spry2*^*−/−*^ mice (*n =* 3). Na^+^ K^+^ ATPase and Tubulin serve as loading controls for membrane and cytosolic fractions, respectively. **(C)** Proximity ligation assay of CSK and Cav-1 (red dots) in US and anti-CD3/CD28 stimulated (10 min) CD4^+^ T cells from *Spry2*^*+/+*^ and *Spry2*^*−/−*^ mice (*n =* 3). Scale bar, 5 μm. **(D, E)** Relative abundance of spots depicted as frequency of cells with spots (% cells with spots) **(D)** and relative frequency of cells with designated number of spots **(E)**. **(F)** Real-time PCR analysis of basal Cav-1 mRNA expression in splenic CD4^+^ T cells from *Spry2*^*+/+*^ and *Spry2*^*−/−*^ mice (*n =* 3). **(G)** Immunoblots of Cav-1, pan-Ub, and actin from CD4^+^ T cells of *Spry2*^*+/+*^ and *Spry2*^*−/−*^ mice cultured for 14 h in MG132 (5 μM) or chloroquine (50 μM) (*n* = 3). **(H)** p-CSK, CSK, p-ERK1/2, and ERK1/2 immunoblots of CD4^+^ T cells from *Spry2*^*−/−*^ mice (*n* = 3) treated with a membrane-permeable AP-ScrP or AP-CSD. **(I, J)** CD4^+^ T cells from *Spry2*^*−/−*^ mice (*n* = 3) treated with a membrane-permeable AP-ScrP or AP-CSD and cultured in CD3/CD28-coated plates for 48 h and analyzed for the release of IL2 and IFNγ. Significance * *p* < 0.05, ** *p* < 0.005, by Student *t* test. All the data of this figure can be found in the S1 and S2 Data files. AP-CSD, antennapedia caveolin scaffolding domain; AP-ScrP, antennapedia scrambled peptide; Cav-1, caveolin-1; IFN-γ, interferon gamma; IL2, interleukin 2; pan-Ub, pan-ubiquitin; Spry2, Sprouty2; US, unstimulated.

Cytosolic CSK requires transmembrane adaptors such as Cbp/PAG-1 and caveolin-1 (Cav-1) for recruitment to lipid rafts and full activation [31–33]. Spry2 deficiency led to a marginal decrease in Cbp/PAG-1 expression and a 4.3-fold higher level of Cav-1 (Fig 8A). Cytosolic and membrane fractions isolated from CD4^+^ T cells showed elevated levels of Cav-1 in the membrane fractions of *Spry2*^*−/−*^ CD4^+^ T cells (Fig 8B). We next examined the association of CSK with Cav-1. We used proximity ligation assay (PLA) to quantify the abundance of CSK–Cav-1 interaction in *Spry2*^*+/+*^ and *Spry2*^*−/−*^ CD4^+^ T cells. Under unstimulated conditions, the abundance of CSK–Cav-1 interaction was higher in *Spry2*^*−/−*^ relative to *Spry2*^*+/+*^CD4^+^ T cells (Fig 8C–8E). Activated CD4^+^ T cells from both *Spry2*^*+/+*^ and *Spry2*^*−/−*^ mice displayed marked reduction in abundance of CSK–Cav-1 interaction. However, the activation-induced attenuation of CSK–Cav-1 interaction was significantly lower in *Spry2*^*−/−*^ when compared to *Spry2*^*+/+*^ CD4^+^ T cells.

The high levels of the Cav-1 protein in *Spry2*^*−/−*^ CD4^+^ T cells was not due to an increase in Cav-1 mRNA expression (Fig 8F), suggesting that Spry2 affected the Cav-1 protein level possibly by regulating the protein turnover. To test this, CD4^+^ T cells from *Spry2*^*+/+*^ and *Spry2*^*−/−*^ mice were pretreated with MG132 (a proteosome inhibitor) and chloroquine (a lysosome inhibitor). Pretreatment with MG132 increased Cav-1 expression in *Spry2*^*+/+*^, but not in *Spry2*^*−/−*^ CD4^+^ T cells. Chloroquine had no effect on Cav-1 degradation in both control and *Spry2*^*−/−*^ CD4^+^ T cells (Fig 8G). These results suggest that Spry2 aids Cav-1 protein degradation by the ubiquitin–proteosome pathway.

The N-terminal domain of Cav-1 contains a caveolin scaffolding domain (CSD, residues 82 to 101), which binds to and serves as a scaffold for several caveolin-interacting proteins including CSK [34,35]. Recombinant CSD peptide fused to Antennapedia homeodomain (AP-CSD) act as a “dominant negative inhibitor” by discharging the interacting proteins from Cav-1 [36]. We hypothesized that disrupting the Cav-1 complex would inhibit CSK activity and enhance ERK1/2 activity in response to TCR stimulation. Treatment of *Spry2*^*−/−*^ CD4^+^ T cells with AP-CSD showed compromised CSK activity (reduced p-CSK levels) and elevated ERK1/2 phosphorylation compared with scrambled peptide treatment (Fig 8H). Similarly, *Spry2*^*−/−*^ CD4^+^ T cells treated with AP-CSD showed significantly elevated levels of both IL2 and IFNγ as compared to *Spry2*^*−/−*^ CD4^+^ T cells treated with control peptide (Fig 8I and 8J). These results suggest that in the absence of Spry2, Cav-1–bound CSK inhibits LCK and diminishes TCR-induced responses in CD4^+^ T cells.

## Discussion

While the role of Spry proteins as negative feedback regulators of receptor tyrosine kinase (RTK) signaling in stromal cells is well established [9,37], very little is known about their role in lymphoid cells. In this study, we show that Spry2 functions as a positive regulator of CD4^+^ T cell function and differentiation by modulating the activity of non-RTKs. Consistent with earlier studies [18,19], we observed that expression of Spry2 is induced upon TCR stimulation, suggesting that Spry2 expression is regulated by TCR at transcriptional level. Our data indicate that Spry2 is an important determinant of effector T cell differentiation as deletion of Spry2 resulted in reduced expression of Th-specific transcription factors and cytokines under Th1-, Th2-, and Th17-skewing conditions. The robust induction of Spry2 in differentiated T effector cells as opposed to metabolically quiescent naïve or memory T cells indicate that Spry2 plays a fundamental role in orchestrating pathways involved in signaling reprogramming that is displayed by T effector cells.

Prior studies suggest that Spry2 inhibits ERK1/2 activation in response to FGF [1,10,37] and potentiates ERK1/2 signaling in response to EGF in a context-dependent manner [38]. Recently, Spry2 was identified as a negative regulator of BCR-mediated ERK1/2 signaling in B cells [16]. In contrast, we show that Spry2 functions as a positive regulator of ERK1/2 signaling in response to TCR stimulation. This differential effect on ERK1/2 signaling by Spry2 suggests diverse upstream mechanisms of activation in different cell types.

An earlier report [39] suggests that ERK signaling pathway stabilizes GATA3 protein levels through ubiquitin–protesome pathway. Further, during Th2 differentiation, IL-4 promotes GATA3 transcription by activating the transcription factor STAT6 [40]. In this study, we show that loss of Spry2 in CD4^+^ T cells causes defective ERK, STAT5, and STAT6 phosphorylation, which could explain defective induction and/or stability of GATA3 protein and type 2 immune responses.

LCK associates with CD4 and plays a central role in initiation of TCR signaling [25]. LCK activation requires an alteration in its conformation. LCK phosphorylation at the C-terminal Y505 residue by CSK results in a closed, inactive conformation, while phosphorylation of Y394 facilitates open conformation and leads to full activation [41]. Spry2 deficiency decreases the activating phosphorylation of Y394 and increases the inhibitory phosphorylation of Y505, suggesting Spry2 antagonizes CSK and prevents Y505 phosphorylation to allow LCK activation. The inability to activate multiple downstream signaling pathways—ERK1/2, and nuclear factor of activated T cells (NFAT)—and produce cytokines belonging to multiple T helper lineages in Spry2-deficient mice is consistent with a failure of the TCR to activate receptor-proximal signaling molecules. An earlier report suggests that ERK1/2 positively regulates the induction of Spry2 [42]. Here, we show a positive correlation between Spry2 induction and ERK1/2 activation in activated CD4^+^ T cells. Thus, we speculate that a dynamic interplay exists between Spry2 induction and ERK1/2 activation in CD4^+^ T cells that leads to optimal TCR-generated signals.

CSK is predominantly cytosolic [29]. Through its SH2 domain, CSK interacts with a number of adapter molecules (e.g., Cbp/PAG1, Caveolin-1, Paxillin, and LIME) which serve as membrane anchors [31–33,43,44]. The association with these adaptor molecules increases CSK activity, thereby enabling inhibitory (Y505) phosphorylation of LCK. Cbp/PAG1 is a major regulator of CSK [31,32]. However, recent studies indicate that Cbp is dispensable for raft localization of CSK in T cells [45]. We show that Spry2 deletion leads to a marginal decrease in Cbp/PAG1 but a robust increase in Cav-1, which results in elevated CSK activity and, consequently, maintenance of LCK in an inhibited state. Functional redundancies exist among Csk-binding proteins as knockdown of either Cav-1 or Cbp results in a compensatory increase in expression of the other to inhibit Src activity [46].

The lipid raft protein, Cav-1, plays an important role in cell migration, endocytosis, and vesicular transport, and regulates a number of signaling pathways via its scaffolding domain [34,47]. Cav-1 also plays an important role in TCR synaptic polarity, signaling strength, T cell proliferation, and cytokine production [48–50]. Cav-1, through its C-terminal and scaffolding domain, interacts with Spry2 and CSK, respectively [51,52]. One of the perturbations in *Spry2*^*−/−*^ T cells was an overabundance of Cav-1 in the membrane, which positively correlated with CSK abundance and activity. Our findings reveal, for the first time, that Spry2 regulates the Cav-1 protein turnover in CD4^+^ T cells by promoting ubiquitination and subsequent proteosomal degradation. Cav-1 is ubiquitinated [53,54], and Spry2, acting as an adaptor, could bring ubiqutin E3 ligases in close proximity to Cav-1 to promote its ubiquitination and degradation. Spry2 interacts with the E3 ubiquitin ligase Cbl [55]. Whether Spry2 directs Cbl to promote Cav-1 ubiquitination is unknown. While it needs to be investigated as to how Spry2 regulates Cav-1 levels, our work does support the notion that the Cav-1–bound CSK plays a major role in defective LCK activation in *Spry2*^*−/−*^ CD4^+^ T cells. A recent study [56] demonstrated that Cav-1 negatively regulated Janus kinase (JAK)/STAT5 signaling pathway. The present study in CD4^+^ T cells from *Spry2−/−* mice showing an up-regulation of Cav-1 and corresponding defective phosphorylation of STAT5 and STAT6, in response to IL-2 and TSLP, suggest that a similar mechanism may exist by which Cav-1, via its scaffolding domain, negatively regulates upstream JAK tyrosine kinases.

This study suggests a novel role for Spry2 as a positive regulator of ERK1/2 signaling and T cell function by modulating LCK activity via CSK. *Spry2*^*−/−*^ mice fail to develop airway hyperreactivity and type 2 inflammation in T cell model of asthma. We employed adoptive transfer of *Spry2*^*−/−*^ T cells to *Rag2*^*−/-*^*γC*^*−/−*^ mice to eliminate the possibility that this defect was due to global Spry2 deletion. Our results demonstrated that Spry2 in CD4^+^ T cells is indispensable for the asthma phenotype. In human T cells, Spry2 knockdown preferentially inhibited type 2 cytokines but not type 1 cytokines. Augmented TNFα production in Spry2 knockdown cells that we observed suggests a differential regulation of human T helper cells and their cytokines by Spry2. Our result is in agreement with a paper by Chiu and colleagues [57], who observed increased TNFα expression in Spry2 knockdown HIV-specific T cells. This was associated with increased polyfunctionality of T cells.

Our study identifies Spry2 as a positive regulator of CD4^+^ T cell function, and type 2 immunity, improving the understanding of Spry2 regulation of TCR signaling. Our results also uncover a novel mechanism of induction of type 2 inflammation in disorders such as asthma.

## Material and methods

### Animals

Mice with Cre recombinase-ERT2 fusion gene driven by ROSA26 promotor (B6.129-*Gt(ROSA)26Sor*^*tm1(cre/ERT2)Tyj*^/J from The Jackson’s Laboratory) and *Spry2*^*f/f*^ (MMRRC, mutant mice research and resource center) were crossed to generate mice carrying ERT2-Cre:*Spry2*^*f/f*^. Global deletion of Spry2 (*Spry2*^*−/−*^) was achieved via intraperitoneal injection of 100 μl of 10 mg/mL Tamoxifen (Sigma, T5648) for 5 consecutive days into 4-week-old ERT2-Cre:Spry2^f/f^ of either sex; mice were rested at least 3 weeks before use in experiments. To genetically deplete Spry2 in CD4^+^ T cells, CD4-Cre mice (Tg(Cd4-cre)1Cwi/BfluJ from The Jackson’s Laboratory) were crossed to *Spry2*^*f/f*^ to obtain CD4-Cre:*Spry2*^*f/f*^ mice. Sex- and age-matched *Spry2*^*f/f*^ mice were used as controls (*Spry2*^*+/+*^) in all our experiments. In addition, 8-week-old *Rag2*^*−/−*^*γC*^*−/−*^ (B10;B6-*Rag2*^*tm1Fwa*^ *Il2rg*^*tm1Wjl*^) female mice were obtained from Taconic (Germantown, New York) and used for adoptive transfer experiments.

### Asthma model

For the *Aspergillus-*driven acute asthma model, female *Spry2*^*+/+*^ and *Spry2*^*−/−*^ mice (*n =* 5) were subcutaneously injected twice with 10 μg *Aspergillus* (Greer Labs, Lenoir, North Carolina, United States of America) or saline suspended in Alum (Imject alum, Thermo Fisher, Rockford, Illinois, USA; Cat# 77161) on day 0 (D0) and day 7 (D7) as described previously [27]. At the indicated times (Fig 3A), mice were intranasally challenged with saline or 10 μg *Aspergillus*, rested for 3 days after the last challenge, and then examined for AHR, lung-infiltrating immune cells, and lung inflammation.

For adoptive transfer experiments, CD4^+^ T cells from *Spry2*^*+/+*^ or *Spry2*^*−/−*^ female mice were sort purified and intravenously transferred to naïve female *Rag2*^*−/-*^*γC*^*−/−*^ (7 × 10^6^ cells in 100 μl saline per mouse) on day 1 (D1). The recipient mice were subcutaneously injected twice with 10 μg *Aspergillus* or saline suspended in Alum on D0 and D7 followed by IN challenge (Fig 4A) and analyses as described above.

### Airway hyperreactivity (AHR) measurement

Three days after the last IN challenge, mice were anesthetized with ketamine (180 mg/kg), xylazine (9 mg/kg), and acepromazine (4 mg/kg), underwent tracheotomy, and were attached via an 18-gauge cannula to small-animal ventilator with computer-controlled piston (Flexivent; Scireq, Montreal, Quebec, Canada). AHR was measured in response to methacholine (Mch) as described previously [27]. Group averages were expressed as the fold increase over baseline resistance (mean ± SEM). Statistical significance was determined using ANOVA and unpaired Student *t* test.

### Histology and morphometric analysis

Formalin-fixed and paraffin-embedded lung tissue sections (5 μm) were stained with hematoxylin and eosin (H&E) for morphometric analysis, periodic acid–Schiff (PAS) for mucin staining, and Masson trichrome for collagen deposition and mounted using permount medium (Thermo Fisher Scientific). Images were acquired on a Nikon Eclipse TE2000-U microscope using ×20 dry lenses. Inflammation, PAS, and Trichrome staining intensity was quantified with Metamorph image acquisition and analysis software (Molecular Devices, Eugene, Oregon). Airway inflammation was measured as the ratio of the total area of peribronchial and perivascular inflammatory infiltrates over the perimeter of the associated basement membrane obtained from a minimum of 5 airways per mouse and 5 mice per group.

### *Aspergillus*-specific IgE

Briefly, *Aspergillus* extract-coated ELISA plates (10 μg/mL) were washed with PBS-Tween (0.05%) and blocked with 10% FBS in PBS for 1 h at room temperature (RT). Triplicate 50 μL volumes of serum from each mouse were then incubated for 2 h at RT. Plates were washed 5 times in PBS and incubated with 1:1,000 biotinylated anti-IgE (BD PharMingen, San Jose, California, United States of America). Plates were then washed and incubated with streptavidin–horseradish peroxidase (BD PharMingen) for 1 h at RT. Plates were washed 7 times and developed with TMB substrate (Pierce) for 20 min. 2N H_2_SO_4_ was added to stop the reaction and the optical density (OD) read at 450 nm. Results are reported as raw OD values.

### Flow cytometry

One million cells were incubated with 1 μg of purified anti-mouse CD16/32 (clone: 93, Biolegend, San Diego, California) in FACS buffer prior to staining. Cell staining was performed with fluorescently labeled anti-mouse monoclonal antibodies (described below) for 30 min at 4°C. Cells were washed with FACS buffer, fixed with 4% paraformaldehyde for 15 min at 4°C, and data acquired using either LSRII or LSRFortessa (BD Biosciences, San Jose, California) flow cytometer. For intracellular staining, cells were incubated for 4 h with monensin (2 μmol/L). Surface staining was performed as above. Cells were then washed in FACS buffer, fixed, and permeabilized (Foxp3 kit, eBioscience, San Diego, California; #00-5523-00) for 1 h at RT. Cell were washed and stained with antibodies against intracellular proteins (cytokines, transcription factors) for 30 min at 4°C. Cells were washed and data acquired using either LSRII or LSRFortessa. All data were analyzed using FlowJo v10 software. Anti-mouse antibodies used for surface and intracellular staining were given in S2 Table.

### Real-time PCR

To detect mouse Cav-1 and Spry2, total RNA from CD4^+^ T cells was extracted using RNeasy mini kit and was reverse transcribed into cDNA with Superscript III first-strand synthesis kit as per manufacturer’s instructions. Gene-specific PCR products were amplified by using qPCR SYBR Green Rox mix. The copy number of Cav-1 and Spry2 was normalized to that of GAPDH by using the 2 ^−ΔΔCt^ method. The following primers were used: Cav-1 –forward, 5′-CTTCGGCATCCCAATGGCACTC-3′ and reverse, 5′-AGGTATGGACGTAGATGGAGTA-3′; Spry2—forward, 5′-TGAAAGACTCCACGGTCTGC -3′ and reverse 5′-AGCTGACAGTGCTGATGGAC-3′; and GAPDH–forward, 5′-CTTTGTCAAGCTCATTTCCTGG-3′ and reverse, 5′-TCTTGCTCAGTGTCCTTGC-3′. Real-time PCR was performed with the ABI Prism 7000 Sequence Detection System (Applied Biosystems, Foster City, California).

### CD4^+^ T cell isolation and differentiation

CD4^+^ T cells were purified from spleens of *Spry2*^*+/+*^ or *Spry2*^*−/−*^ mice using mouse naïve CD4^+^ T cell isolation kit (Cat# 19765; Stem Cell Technologies, Vancouver, British Columbia, Canada). Flow cytometry confirmed purity in excess of 97%. Sort-purified naïve CD4^+^ T cells (10^6^ cells/well) were seeded on plate bound anti-CD3 (1 μg/mL, clone: 145-2C11, Biolegend, Cat# 100302) and anti-CD28 (1 μg/mL, clone: 37.51, Biolegend, Cat# 102102) antibodies and cultured for 3 days with murine IL-2 (mIL-2, 20 ng/mL, Peprotech, Cranbury, New Jersey; Cat# 212–12) under the following conditions: Th0, anti-IFN-γ (10 μg/mL clone: XMG1.2, Biolegend) and anti-IL-4 mAbs (10 μg/mL clone 11B11, Biolegend); Th1, recombinant mouse IL-12 (10 ng/mL, R&D Systems, Minneapolis, Minnesota) and anti-mouse IL-4 mAb; Th2, recombinant mouse IL-4 (10 ng/mL, R&D Systems) and anti-IFN-γ and anti-mouse IL-12 mAbs (10 μg/ml, clone C15.6, BD Biosciences); Th17, recombinant human TGFβ (3 ng/mL; Peprotech), recombinant mouse IL-6 (20ng/mL, R&D Systems) anti-IFN-γ, anti-IL-4, and anti-mouse IL-12 mAbs. Cells were then rested for 3 additional days in fresh media with IL-2, harvested, and analyzed.

### CD4^+^ T cell proliferation assay

CD4^+^ T cells (10^6^ cells/well) from *Spry2*^*+/+*^ or *Spry2*^*−/−*^ mice were cultured for 3 days on plates coated with anti-CD3 (2 μg/mL), anti-CD3/CD28 (2 μg/mL each), or anti-CD3/CD28 + mIL-2 (20 ng/mL; Peprotech). Cells were labeled with CFSE (200 nM) and proliferation analyzed by flow cytometry. Human CD4^+^ T cell proliferation as measured by [^3^H]-Thymidine incorporation was performed as previously described [58].

### Western blotting

Purified splenic CD4^+^ T cells from *Spry2*^*+/+*^ or *Spry2*^*−/−*^ mice were resuspended in complete RPMI1640 and stimulated with plate-bound anti-CD3 (10 μg/mL) and anti-CD28 (2 μg/mL) for 0, 5, 10, and 30 min. Activated CD4^+^ T cells were washed with ice-cold PBS and lysed in RIPA buffer with protease and phosphatase inhibitor cocktail (Sigma, St. Louis, Missouri). Cytosol and membrane fractions of CD4^+^ T-cells were also isolated by Subcellular Protein Fractionation Kit (Thermo Fisher, Cat#78840).

Equal amounts of protein were loaded on SDS-PAGE, transferred on to Nitrocellulose membrane (GE, Amersham). Blots were subsequently blocked with 5% Blotto in TBS-T for 1 h at RT, followed by overnight incubation with primary antibodies. After 3 washes with TBS-T (5 min each), blots were incubated in HRP-conjugated secondary Abs for 1 h at RT, followed by 3 washes with TBS-T. Pierce ECL western blotting substrate was used to visualize bands, and the bands were quantified using the ImageJ software. The primary antibodies used for western blotting were given in S2 Table.

### Immunoprecipitation

Coimmunoprecipitation was done as described previously [59]. Briefly, CD4^+^ T cells were washed with ice-cold PBS and lysed in buffer containing 25 mM Tris-HCl (pH7.4), 150 mM NaCl, 1.0% TritonX-100, 5 mM EDTA, 0.1% BSA, and protease and phosphatase inhibitor cocktail (Sigma) at 4°C for 20 min. Lysates were centrifuged at 10,000*g* for 10 min, and supernatants were incubated either with 4 μg of LCK antibody or appropriate control Ig for 12 h at 4°C on a Roto-torque followed by 2 h of incubation with protein A/G plus agarose beads to immunoprecipitate LCK-bound proteins. The bound fraction was washed, and immunoprecipitates were lysed in SDS containing Laemmli sample buffer. The samples were then subjected to western blotting analysis as described above.

For in vitro kinase assay, CD4^+^ T cells (5 × 10^7^) were lysed in Pierce IP lysis buffer (# 87787, Thermo Fisher) containing protease and phosphatase inhibitor cocktail (Sigma) at 4°C for 20 min. Lysates were centrifuged at 10,000*g* for 10 min, and supernatants were incubated either with 4 μg of LCK antibody or appropriate control Ig for 12 h at 4°C on a Roto-torque followed by 2 h of incubation with Protein G Magnetic Beads (#70024, CST, Danvers, Massachusetts). Magnetic bead–antibody complex were washed 4 times with IP lysis buffer and twice with 500 μl 1× kinase buffer (#9802, CST) using a magnetic separation rack. Washed pellets were suspended in 40 μl of 1× kinase buffer supplemented with 200 μM ATP (#9804, CST) and 1 μg of recombinant mouse CSK (#50893-M20B, Sino Biological, Beijing, China) or 1 μg of recombinant mouse CSK with increasing concentrations (0.5 μg or 1 μg or 2 μg) of recombinant mouse Spry2 (MyBiosource, San Diego, California; #MBS1362850). Pellets were incubated for 30 min on a heating block at 30°C, and the kinase reaction was terminated using 4× Laemmli sample buffer. Immunoblot analysis was done as described above.

### Immunohistochemistry (IHC) and immunocytochemistry (ICC)

Formalin-fixed paraffin-embedded lung tissue sections from *Aspergillus*-treated *Spry2*^*+/+*^ and *Spry2*^*−/−*^ (*n =* 3) mice were deparaffinized with Citrisolv, rehydrated in graded alcohol series, and further rehydrated in PBS. To retrieve epitopes for efficient antibody binding, sections were microwaved for 15 min in 10 mM citrate buffer (pH 6.0). Sections were blocked with blocking buffer (3% BSA in PBS) for 1 h at RT and were incubated overnight in a humid chamber at 4°C in primary antibodies CD4 and p-LCKY505 diluted in blocking buffer; followed by 3 washes with PBS. Appropriate fluorescent conjugated secondary antibodies (Molecular Probes, Eugene, Oregon) diluted in blocking buffer (1:200) were added to the sections for 1 h at RT in dark, followed by 3 PBS washes, and mounted using Prolong antifade reagent containing DAPI.

For ICC, CD4^+^ T cells were fixed (2% paraformaldehyde) and cytospun onto charged slides. We followed the same staining procedure as described above, except that slides were not subjected to deparaffinization, rehydration, or heat-induced antigen retrieval steps. However, cells were permeabilized in PBS containing 0.5% TritonX-100 and 0.05% Tween-20 for 5 min before the staining procedure. Images were acquired using Zeiss LSM 700 Confocal microscope with 63× oil immersion objective lens. Images were analyzed and processed using Zen Blue software (Carl Zeiss, Germany) and further processed using Adobe photoshop. Pearson correlation coefficients for colocalization were calculated by Zen Blue software. The details of primary antibodies and the dilutions at which they were used for ICC were given in S2 Table.

### Proximity ligation assay (PLA)

Unstimulated and anti-CD3/CD28-stimulated CD4^+^ T cells were fixed (10 min in 3.7% formaldehyde); cytospun onto charged slides and washed in PBS (2× 5 min each) before permeabilization in PBS containing 0.5% TritonX-100 and 0.05% Tween-20. PLA was done essentially according to manufacturer’s instructions provided along with the kit (Duolink In Situ Red Starter Kit Mouse/Rabbit; Cat# DUO92101). Briefly, slides were incubated in blocking buffer for 1 h at RT and further incubated overnight in primary antibodies (mouse CSK and rabbit Cav-1) diluted in appropriate buffer at 4°C. Slides were then rinsed 2× 5 min in wash buffer, incubated in PLA probes for 1 h at 37°C, washed again (2× 5 min), and incubated in ligase for 30 min at 37°C before proceeding to amplification step. Finally, slides were washed and mounted using Duolink In Situ Mounting Medium with DAPI; images were acquired using Zeiss LSM 700 Confocal microscope and analyzed by Zen Blue software.

### Human CD4^+^ T cell isolation and ELISA

Peripheral blood mononuclear cells (PBMCs) were isolated by density gradient centrifugation using Histopaque (Sigma). CD4^+^ T cells were purified from PBMCs by negative selection using CD4^+^ T cell isolation kit (Miltenyi Biotech, Germany). Sort-purified T cells were transduced with Lentiviral vectors harboring either Control shRNA (shLuc) or SPRY2 shRNA (transOMIC Technologies, Huntsville, Alabama, USA) as described below. Cells were cultured under T helper differentiation conditions for 6 days, supernatants were collected and analyzed by ELISA for human TNF-α (Biolegend), IL-4 (R&D Systems), IL-10, IL-17, and IFN-γ (eBioscience) according to manufacturer’s instructions. BALF collected from saline or Asp-treated *Spry2*^*+/+*^ *and Spry2*^*−/−*^ mice were analyzed for IL5 and IL13 using Duoset ELISA kits according to manufacturer’s instructions.

### Lentiviral transduction

The lentiviral packaging plasmid psPAX2 (#12260) and the envelope plasmid pMD2G (#12259) were purchased from Addgene (Massachusetts, USA). shERWOOD UltramiR Lentiviral vectors that harbored a short hairpin RNA sequence against mouse CSK and encoded ZsGreen (GFP) (CSK shRNA) and corresponding control lentiviral vectors that encoded ZsGreen (Con shRNA) were obtained from transOMIC Technologies (Huntsville, Alabama, USA). The viral packaging cell line LentiX293T was purchased from Takara (Mountain View, California) and maintained in complete DMEM. To generate high-titer lentiviral particles, LentiX293T cells were plated in 60 mm tissue culture dishes until they reached 70% confluency. LentiX293T cells were then cotransfected with 2 μg of psPAX2 + pMD2G (1:3 ratio) along with 2 μg of plasmids containing ConshRNA or CSK shRNA1or CSK shRNA2 or CSK shRNA3 using Lipofectamine2000. Supernatant was collected at 24 and 48 h post transfection and passed through 0.45 μm syringe filter to remove cell debris. Sort-purified CD4^+^ T cells from *Spry2*^*+/+*^ and *Spry2*^*−/−*^ mice were plated in anti-CD3/CD28-coated 12-well plates (2 × 10^6^ cells/well) 24 h prior to viral transduction. Following day, CD4^+^ T cells were infected with viral particles containing either Con shRNA or CSK shRNA along with 10 μg/mL of polybrene (Millipore, Burlington, Massachusetts) and 20 ng/mL of mIL-2 (Peprotech).

### Peptides

Peptides corresponding to either CSD (N-terminal cytoplasmic region of Cav-1 with amino acids 82–101; DGIWKASFTTFTVTKYWFYR) or the scrambled control (WGIDKASFTTFTVTKYWFRY; fused at the N-terminus to the cell-permeable Antennapedia internalization sequence (43–58) RQIKIWFQNRRMKWKK) were purchased from Sigma (Cat# 219482 and 219483). Lyophilized peptides were resuspended in DMSO at 10 mM, then diluted to 1 mM in distilled water. CD4^+^ T cells from *Spry2*^*−/−*^ mice (10^6^ cells/group) were treated with CSD or scrambled control peptides (10 μM) for 8 h and subsequently remained unstimulated or were stimulated with plate-bound anti-CD3/CD28 for 10 min. Cells were then analyzed by western blotting.

### Statistics

All statistical analyses were performed using GraphPad Prism 7 software. Statistical significance in human studies was calculated by the nonparametric Mann–Whitney *U* test. Statistical analyses in mouse studies were done by Student *t* test. Results are shown as mean +/− SEM. All in vitro experiments were repeated independently at least 3 times. For all analyses, significance was determined at *: *p* < 0.05, **: *p* < 0.005, ***: *p* < 0.0001.

### Ethics statement

All experiments involving mice were carried out in accordance with protocols approved by National Jewish Health Institutional Animal Care and Use Committee (Protocol # AS2614).

Whole blood and bronchoalveolar lavage (BAL) fluid was from patients with allergic asthma, disease control, and healthy donors. Patients with allergic asthma and disease control were recruited from outpatient clinics at National Jewish Health. Bronchoscopy and BAL were performed concurrent with clinical work-up. None of the disease control patients met the ATS diagnostic criteria for asthma. Protocols for blood and BAL studies of lymphocytes from patients with asthma and disease control were approved by the Institutional Review Board (Protocol #: HS-1700). Healthy donors were recruited from the blood bank of National Jewish Health. Patients with asthma and disease control maintained their controller medications at the time of bronchoscopy. Demographic and clinical characteristics of patients with asthma and disease control are shown in S1 Table.

## Supporting information

S1 Table List of demographic and clinical characteristics of the study patients. (PDF)

S2 Table List of antibodies and reagents used in the study. (PDF)

S1 Data The raw data associated with all graphs in the manuscript. (XLSX)

S2 Data Raw images of original unedited western blots shown in the main figures and supporting information. Fig 1A. Western blot of spleen CD4^+^ T cells from Tamoxifen-treated Spry2^f/f^ (*Spry2*^*+/+*^) and ERT^2^-Cre:Spry2^f/f^ (*Spry2*^*−/−*^) mice for Spry2 and actin Fig 1H. Western blot showing Cleaved Caspase-3 (CC-3) levels in splenic CD4+ T cells from *Spry2*^*+/+*^ and *Spry2*^*−/−*^ mice. Fig 2A. Immunoblot of basal Spry2 and GAPDH expression in thymocytes (Thy), splenocytes (Spl), naïve (Tn), and memory T cells from B6 mice. Fig 2C. Immunoblot of ERK activation, Spry2 protein induction, and actin expression in unstimulated (US) and stimulated (CD3 and CD3/28) human CD4^+^ T cells at indicated time points (24 h and 48 h, respectively); P/I represents phorbol myristate acetate and ionomycin. Fig 2G. Immunoblot of Spry2 and transcription factor (T-bet, GATA3, and RORgT) expression in naïve and differentiated CD4^+^ T cells from C57BL/6 mice (*n =* 3). Fig 2H. Immunoblot of T-bet, GATA3, and RORγT in CD4^+^ T cells from *Spry2*^*+/+*^ and *Spry2*^*−/−*^ mice. Fig 2J. Immunoblot of p-STAT5 and p-STAT6 in CD4^+^ T cells, treated with IL-2 (10 ng/mL) and TSLP (10 ng/mL) at indicated time points (in min). * in the blot indicates the location of p-STAT6/STAT6 bands. Fig 6A. p-Tyr and β-Actin immunoblots of sort-purified, anti-CD3/CD28-stimulated CD4^+^ T cells from *Spry2*^*+/+*^ and *Spry2*^*−/−*^ mice. Fig 6B. LCK, ZAP70, CD3ζ, and ERK immunoblots of sort-purified, anti-CD3/28-stimulated CD4^+^ T cells from *Spry2*^*+/+*^ and *Spry2*^*−/−*^ mice. * indicates the location of p-CD3ζ bands. Fig 6F and 6G. p-ERK1/2 and ERK1/2 immunoblots of CD4^+^ T cells from *Spry2*^*+/+*^ and *Spry2*^*−/−*^ mice stimulated with anti-CD3/CD28 or PMA/Ionomycin for 10 min (F) or recombinant IL-33 (20 ng/mL) for 30 min (G). Fig 7C. Immunoblot analysis of total CSK, actin, and Spry2 levels in *Spry2*^*+/+*^ and *Spry2*^*−/−*^ CD4^+^ T cells. Fig 7D. p-CSK and CSK immunoblots of cytosol and membrane fractions of stimulated CD4^+^ T cells from *Spry2*^*+/+*^ and *Spry2*^*−/−*^ mice. Na^+^ K^+^ ATPase and Tubulin serve as loading controls for membrane and cytosolic fractions, respectively. Fig 7E. Immunoprecipitation of LCK and immunoblotting of LCK, CSK, and p-CSK in CD4^+^ T cells from *Spry2*^*+/+*^ and *Spry2*^*−/−*^ mice under unstimulated and anti-CD3/CD28-stimulated (10 min) conditions. Fig 8A. Immunoblot of basal Cav-1, Cbp/PAG-1, GAPDH, and Spry2 levels in CD4^+^ T cells from *Spry2*^*+/+*^ and *Spry2*^*−/−*^ mice. Fig 8B. Immunoblots of Cav-1 from cytosol and membrane fractions of stimulated CD4^+^ T cells from *Spry2*^*+/+*^ and *Spry2*^*−/−*^ mice. Na^+^ K^+^ ATPase and Tubulin serve as loading controls for membrane and cytosolic fractions, respectively. Fig 8G. Immunoblots of Cav-1, pan-ubiquitin (pan-Ub), and actin from CD4^+^ T cells of *Spry2*^*+/+*^ and *Spry2*^*−/−*^ mice cultured for 14 h in MG132 (5 μM) or chloroquine (50 μM). Fig 8H. p-ERK1/2 and ERK1/2, p-CSK, and CSK immunoblots of CD4^+^ T cells from *Spry2*^*−/−*^ mice treated with a membrane permeable scrambled peptide (AP-ScrP) or Caveolin Scaffolding Domain (AP-CSD). S3A Fig. An immunoblot showing lentiviral-mediated knockdown of endogenous CSK and actin in CD4^+^ T cells from B6 mice. S3B Fig. Immunoblot of TCR-driven ERK1/2 phosphorylation in CD4^+^ T cells from *Spry2*^*+/+*^ and *Spry2*^*−/−*^ mice transduced Control shRNA (Con shRNA), CSK shRNA1, or CSK shRNA3. S4B Fig. Immunoprecipitation of LCK from murine CD4^+^ T cells followed by a kinase assay in the presence of recombinant mouse CSK (r-CSK; Lanes: 4, 5, 6, 7 of the Ponceau stained blot) or recombinant mouse Spry2 (r-Spry2; Lanes: 5, 6, 7); WCL represents 5% total cell lysate; IgH and IgL represent Ig heavy and light chains, respectively. Fig 1C and 1E. Gating strategy for the flow cytograms presented in Fig 1C and 1E. Fig 5A. Gating strategy for the flow cytograms for CD4^+^ T cells from blood PBMCs and bronchoalveolar lavage (BAL) FCS files for Figs 1C, 1E, 1G, 1I, 1J, 1K and 1L and 2D and 2F and S1B, S1C and S1E, S2F and S3 (representing the gating strategy for the cited flow plots), and FCS files for Fig 5A (gating strategy for human CD4+ Spry2+ T cells) are shown in the flow repository database (ID: FR-FCM-Z3G3).
(PDF)

S1 Fig Effect of CD4-targeted Spry2 deletion. **(A)** Immunoblot analysis of CD4^+^ T cells and B cells from *Spry2*^*f/f*^ and *CD4-Cre*:*Spry2*^*f/f*^ *mice* (*n =* 2) confirm CD4^+^ T cell–specific deletion of Spry2. **(B)** Splenic CD4^+^ and CD8^+^ T cell frequencies from *Spry2*^*f/f*^ and *CD4-Cre*:*Spry2*^*f/f*^ *mice* (*n* = 3). **(C)** Proliferation of anti-CD3/CD28- or anti-CD3/28+IL-2–stimulated CD4^+^ T cells from *Spry2*^*f/f*^ and *CD4-Cre*:*Spry2*^*f/f*^ mice (*n* = 3). **(D)** Immunoblots of TCR-driven ERK1/2 phosphorylation in CD4^+^ T cells from *Spry2*^*f/f*^ and *CD4-Cre*:*Spry2*^*f/f*^ mice (*n* = 3). **(E)** Intracellular IFN-γ and IL-4 frequencies in CD4^+^ T cells from *Spry2*^*f/f*^ and *CD4-Cre*:*Spry2*^*f/f*^ mice under Th-skewing conditions (*n* = 3). **(F)** Representative images of H&E staining for lung tissue inflammation obtained from Sal or OVA-treated *Spry2*^*f/f*^ and OVA-treated *CD4-Cre*:*Spry2*^*f/f*^ mice. Scale bar, 100 μm. **(G)** Inflammatory area per μm of bronchial BM *** *p* < 0.0001 (*n* = 5 mice/group). All the data of this figure can be found in the S1 Data file. BM, basement membrane; ERK1/2, extracellular signal-regulated kinase 1/2; H&E, hematoxylin and eosin; IFN-γ, interferon gamma; OVA, ovalbumin; Sal, saline; Spry2, Sprouty2; TCR, T cell receptor.
(TIF)

S2 Fig Effect of Spry2 deletion on T helper cell differentiation. **(A)** Frequency of cytokine (IFN-γ, IL-4, and IL-17A)+ *Spry2+/*+ and *Spry2−*/− CD4 T cells cultured under Th1, Th2, and Th17 conditions as determined by flow cytometry (*N =* 3). **(B)** A flow cytogram depicting the surface expression of CD25 in splenic CD4^+^ T cells from *Spry2*^*+/+*^ and *Spry2*^*−/−*^ mice. All the data of this figure can be found in the S1 Data file. IFN-γ, interferon gamma; Spry2, Sprouty2.
(TIF)

S3 Fig Knockdown of CSK in *Spry2*^*−/−*^ CD4^+^ T cells restores ERK1/2 phosphorylation, proliferation, and cytokine production. **(A)** An immunoblot showing lentiviral-mediated knockdown of endogenous CSK in CD4^+^ T cells from B6 mice. Numerical values indicate relative densitometric quantification of CSK normalized to actin. **(B)** Immunoblot of TCR-driven ERK1/2 phosphorylation in CD4^+^ T cells from *Spry2*^*+/+*^ and *Spry2*^*−/−*^ mice transduced Con shRNA, CSK shRNA1, or CSK shRNA3. Numerical values indicate relative densitometric quantification of p-ERK1/2 normalized to total ERK1/2. **(C–E)** Proliferation as assessed by Ki67 staining (*n =* 3) and IFNγ (*n* = 3) and IL-4 secretion (*n* = 5) of GFP^+^ anti-CD3/CD28-stimulated (2 μg/mL each for 48 h) CD4^+^ T cells from *Spry2*^*+/+*^ and *Spry2*^*−/−*^ mice transduced with GFP-expressing Con shRNA, CSK shRNA1, or CSK shRNA3. Significance * *p* < 0.05; **p < 0.005 by Student *t* test. All the data of this figure can be found in the S1 and S2 Data files. Con shRNA, Control shRNA; ERK1/2, extracellular signal-regulated kinase 1/2; GP, green fluorescent protein; IFN-γ, interferon gamma; NTC, nontransduced CD4^+^ T cells; TCR, T cell receptor.
(TIF)

S4 Fig Spry2 colocalizes with CSK in the lipid rafts of CD4^+^ T cells. **(A)** Representative confocal images of CTX-B, CSK, and Spry2-stained, DAPI-counterstained anti-CD3/28-stimulated CD4^+^ T cells from B6 mice (*n* = 3). Scale bar, 5 μm. Colocalization shown in merged images. (**B**) Immunoprecipitation of LCK from murine CD4^+^ T cells followed by a kinase assay in the presence of r-CSK (Lanes: 4, 5, 6, 7 of the Ponceau stained blot) or r-Spry2 (Lanes: 5, 6, 7); WCL represents 5% total cell lysate; IgH and IgL represent Ig heavy and light chains, respectively. All the data of this figure can be found in the S2 Data file. CTX-B, Cholera Toxin-B; Ig, immunoglobulin; r-CSK, recombinant mouse CSK; r-Spry2, recombinant mouse Spry2; Spry2, Sprouty2; WCL, whole cell lysate.
(TIF)

## Abbreviations:

AHR: airway hyperresponsiveness
Asp: Aspergillus
BAL: bronchoalveolar lavage
BALF: bronchoalveolar lavage fluid
Bcl-2: B cell lymphoma 2
BCR: B cell receptor
Cav-1: caveolin-1
CFSE: carboxyfluorescein succinimidyl ester
ConSh: control ShRNA
CSD: caveolin scaffolding domain
CTX-B: Cholera Toxin-B
DP: double-positive
EGF: epidermal growth factor
ERK1/2: extracellular signal-regulated kinase 1/2
FGF: fibroblast growth factor
GFP: green fluorescent protein
H&E: hematoxylin and eosin
IN: intranasal
JAK: Janus kinase
NFAT: nuclear factor of activated T cells
OD: optical density
PAS: periodic acid–Schiff
PLA: proximity ligation assay
PMA: phorbol myristate acetate
RT: room temperature
RTK: receptor tyrosine kinase
shRNA: short hairpin RNA
ShSPRY2: Spry2 ShRNA
SP: single-positive
Spry2: Sprouty2
Tn: naïve CD4^+^ T cell
TNF-α: tumor necrosis factor alpha
TSLP: thymic stromal lymphopoietin
