# Supplementary material for: Sprouty2 positively regulates T cell function and airway inflammation through regulation of CSK and LCK kinases
Source: PLoS Biol. 2021 Mar 8;19(3):e3001063. doi: 10.1371/journal.pbio.3001063 (PMC7971865; doi:10.1371/journal.pbio.3001063)
Supplement: S1 Table — (PDF) [file pbio.3001063.s001.pdf]

S1Table. Demographic and clinical characteristics of the study patients

Parameters	Asthmatic patients	Disease controls
N	8	8
Diagnoses	8 patients with asthma; 8 patients with allergic rhinitis, 7 with chronic sinusitis, 6 with GERD, 1 with bronchiectasis and 1 with aspiration	8 patients with chronic cough and concurrent allergic rhinitis & GERD, 1 patients with bronchiectasis, 2with chronic aspiration and 1 with COPD
Male/female	3/5	4/4
Age	53.2 ± 4 (53)	51.7 ± 4 (53)
FEV1 (%)	72 ± 4 (72^{α})*	88.7 ± 3 (89)
Reversibility (%)	15.0 ± 6 (13)*	3.0 ± 0.7 (1)
PC20 (mg/ml) for methacholine	2.8 ± 0.5 (2.1)*a	23.8 ± 0.7 (24)
Allergy skin test positivity	8	8
Eosinophils /µL blood	490 ± 88 (300)*	100 ± 22 (100)
Total IgE (KIU/L)	279 ± 45 (178)	154 ± 72 (59)
BMI	28.1 ± 2 (28)	27.8 ± 1 (27)
Asthma medications	systemic steroids: 2 patients<br>Omalizumab: 2<br>ICS/LABA: 8<br>LTI: 2<br>SABA: 8<br>Tiotropium: 2	ICS/LABA: 3<br>SABA: 6

FEV1: Forced expiratory volume in the 1st second; PC20: Provocation concentration needed to induce a 20% drop in FEV1; BMI: Bone mass index; GERD: Gastroesophageal reflux disease; ICS: inhaled corticosteroid; LABA: Long-acting beta agonist; SABA: Short-acting beta agonist; LTI: leukotriene inhibitor;

α: number in the parenthesis indicates median; *: $P<0.05$, Mann-Whitney U test; a: performed with 35 patients
