# Supplementary material for: Sprouty2 positively regulates T cell function and airway inflammation through regulation of CSK and LCK kinases
Source: PLoS Biol. 2021 Mar 8;19(3):e3001063. doi: 10.1371/journal.pbio.3001063 (PMC7971865; doi:10.1371/journal.pbio.3001063)
Supplement: S2 Table — (PDF) [file pbio.3001063.s002.pdf]

Supplementary Table: 2

Reagent List:

**Flowcytometry**	Antibody	Clone	Cat#	Vendor
	CD4	GK1.5	100428	Biolegend
	CD8	53-6.7	100725	Biolegend
	Bcl2	BCL/10C4	633508	Biolegend
	Ki67	16A8	652404	Biolegend
	IL-2	JES6-5H4	503808	Biolegend
	IL-4	11B11	504104	Biolegend
	IFN-γ	XMG1.2	505850	Biolegend
	IL-17A	TC11-18H10.1	506904	Biolegend
	hCD4	OKT4	317410	Biolegend
	p-ERK1/2	4B11B69	675504	Biolegend
	Annexin-V		550474	BD
	Spry2		ab60719	Abcam
	CD25	PC61	102026	Biolegend

Immunoblotting

Antibody	Cat#	Vendor
Spry2	14954	CST
GATA3	5852	CST
p-LCK Y505	2751	CST
p-ERK	8544	CST
ERK	4696	CST
Caveolin-1	3267	CST
Na^+K^+ ATPase	3010	CST
GAPDH	3683	CST
Actin	12262	CST
Tubulin	5346	CST
p-Tyr	9411	CST
Ubiquitin	14049	CST
p-STAT5	9359	CST
STAT5	94205	CST
p-STAT6	56554	CST
STAT6	5397	CST
T-bet	644801	Biolegend
RORγT	14-6980-80	eBioscience
p-CSK S364	orb156471	Biorbyt
CSK	sc166560	Santa Cruz Biotech
Cbp/PAG-1	ab155100	Abcam
p-LCK Y394	SAB4300118	Sigma
LCK	SAB2502039	Sigma
p-ZAP70	2701	CST
ZAP70	32760	Santa Cruz
p-CD3ζ	SAB4301233	Sigma
CD3ζ	sc-1239	Santa Cruz

IHC and IF

Antibody	Clone	Cat#	Vendor	Dilution
CD4	4SM95	14-9766-82	eBioscience	1/10
NFATc2		sc-7296	Santa Cruz Biotech	1/100
CSK		sc- 166560	Santa Cruz Biotech	1/100
LCK		sc-13	Santa Cruz Biotech	1/100
Sprouty2		orb103535	Biorbyt	1/100

CholeraToxinB	C-34778	Molecular probes	1/100
Caveolin-1	3267	CST	1/200
p-LCK Y505	ABIN743588	antibodies-online	1/100
