# Supplementary material for: Sprouty2 positively regulates T cell function and airway inflammation through regulation of CSK and LCK kinases
Source: PLoS Biol. 2021 Mar 8;19(3):e3001063. doi: 10.1371/journal.pbio.3001063 (PMC7971865; doi:10.1371/journal.pbio.3001063)

Full unedited Blot for Figure 1A

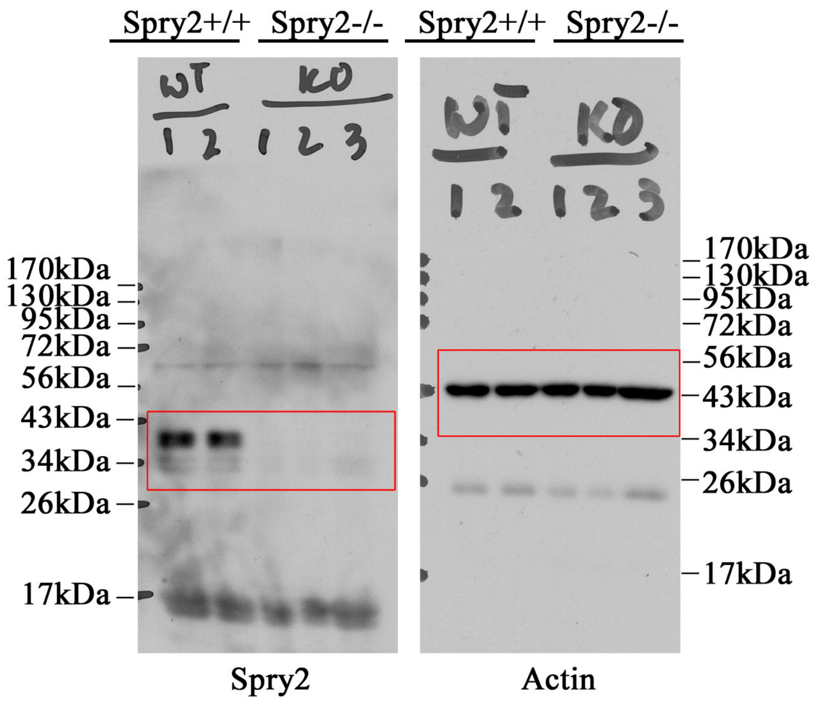

Full unedited Blot for Figure 1H

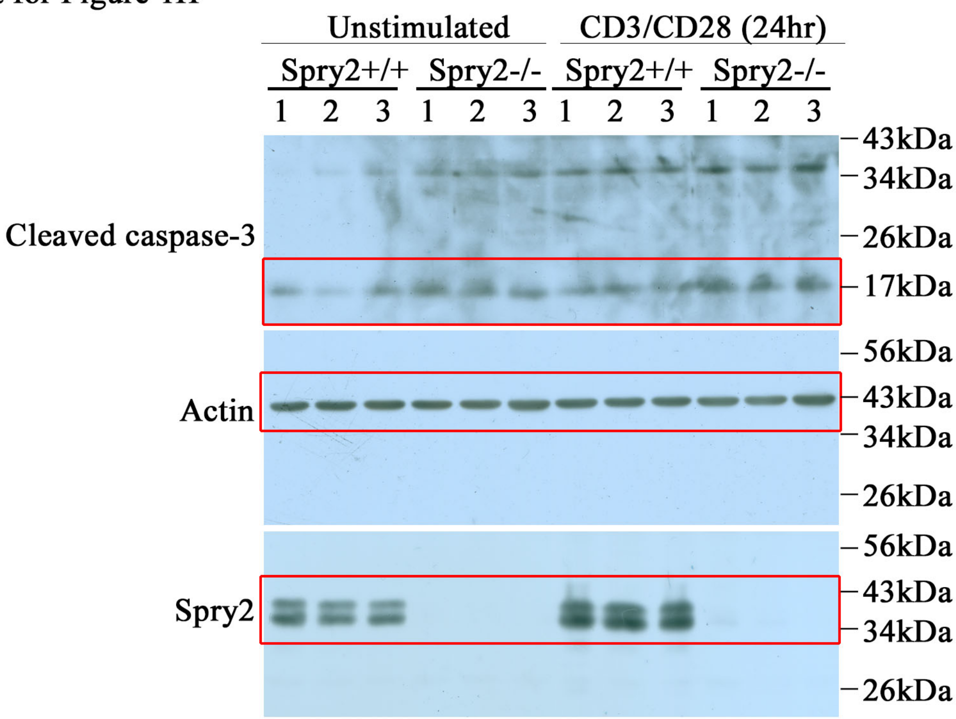

Full unedited Blot for Figure 2 A and 2 E

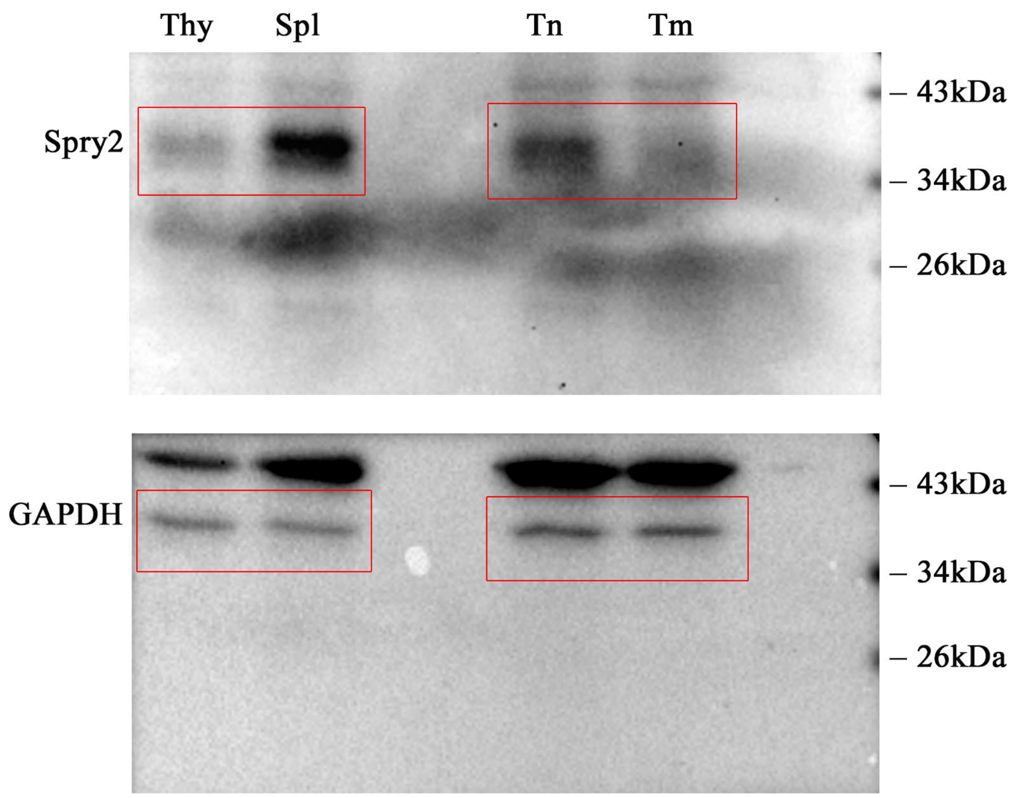

Full unedited Blot for Figure. 2 C

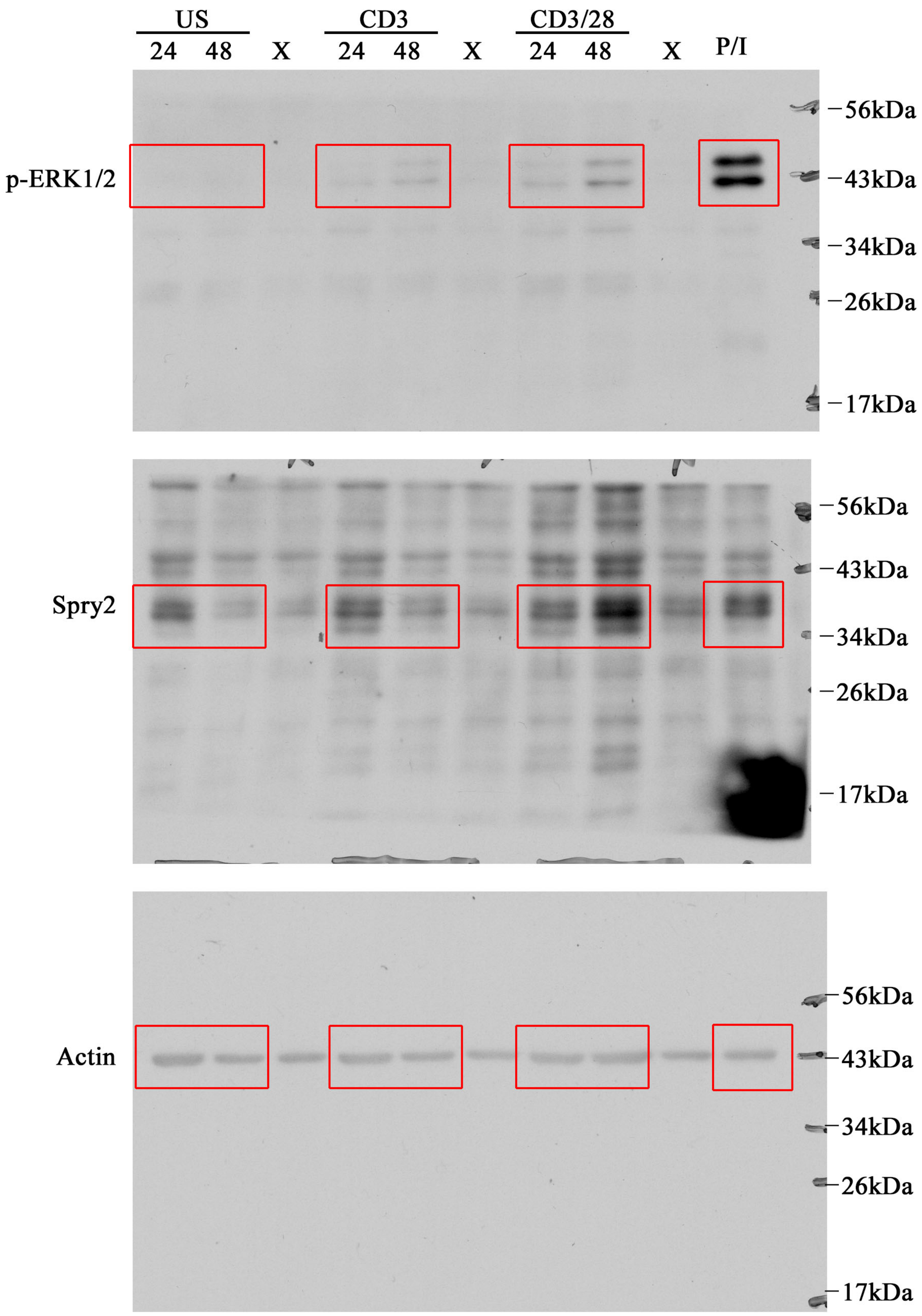

Full unedited Blot for Figure 2G

Blots here were probed with ECL reagent and images are aquired using BioRad ChemiDoc

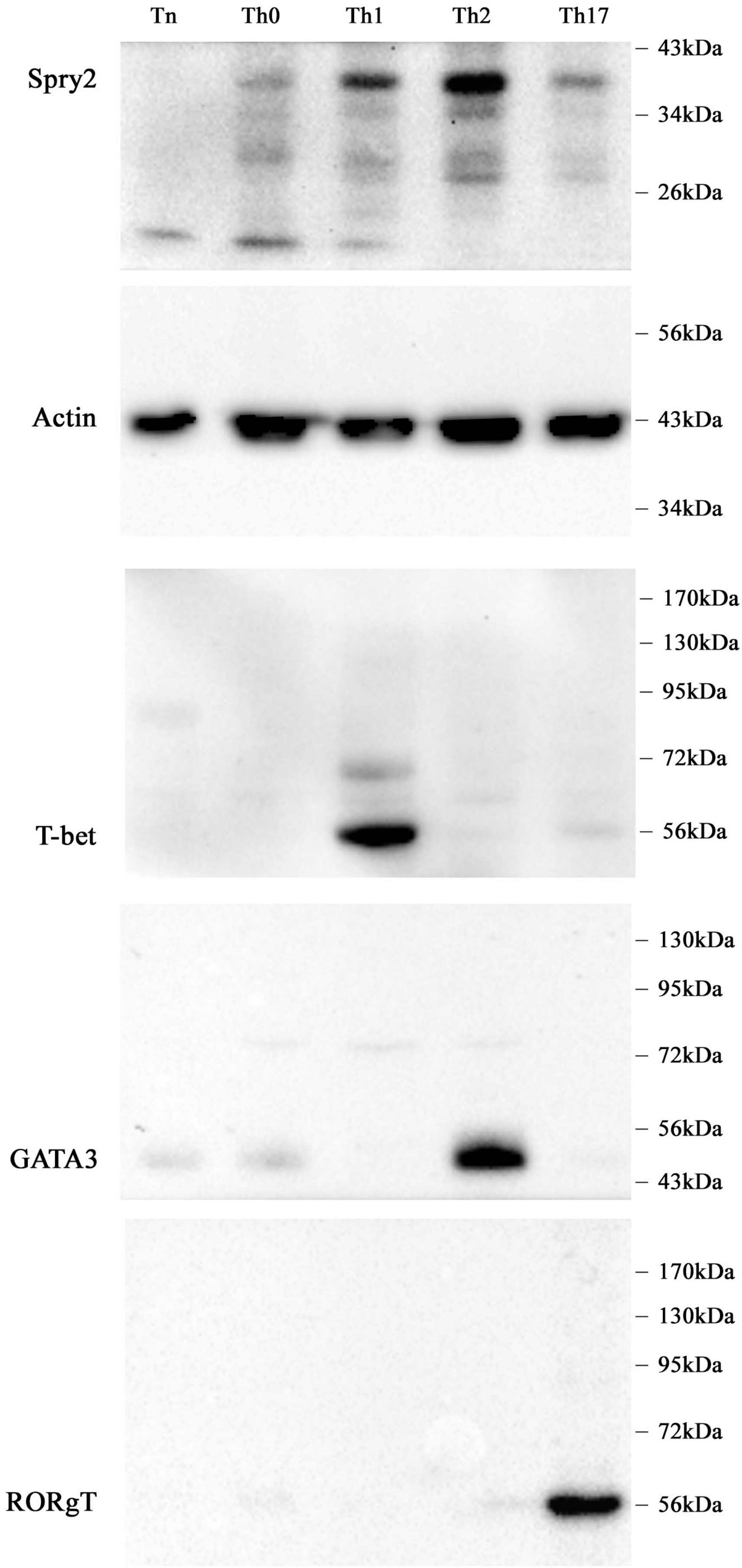

Full unedited Blot for Figure 2 H

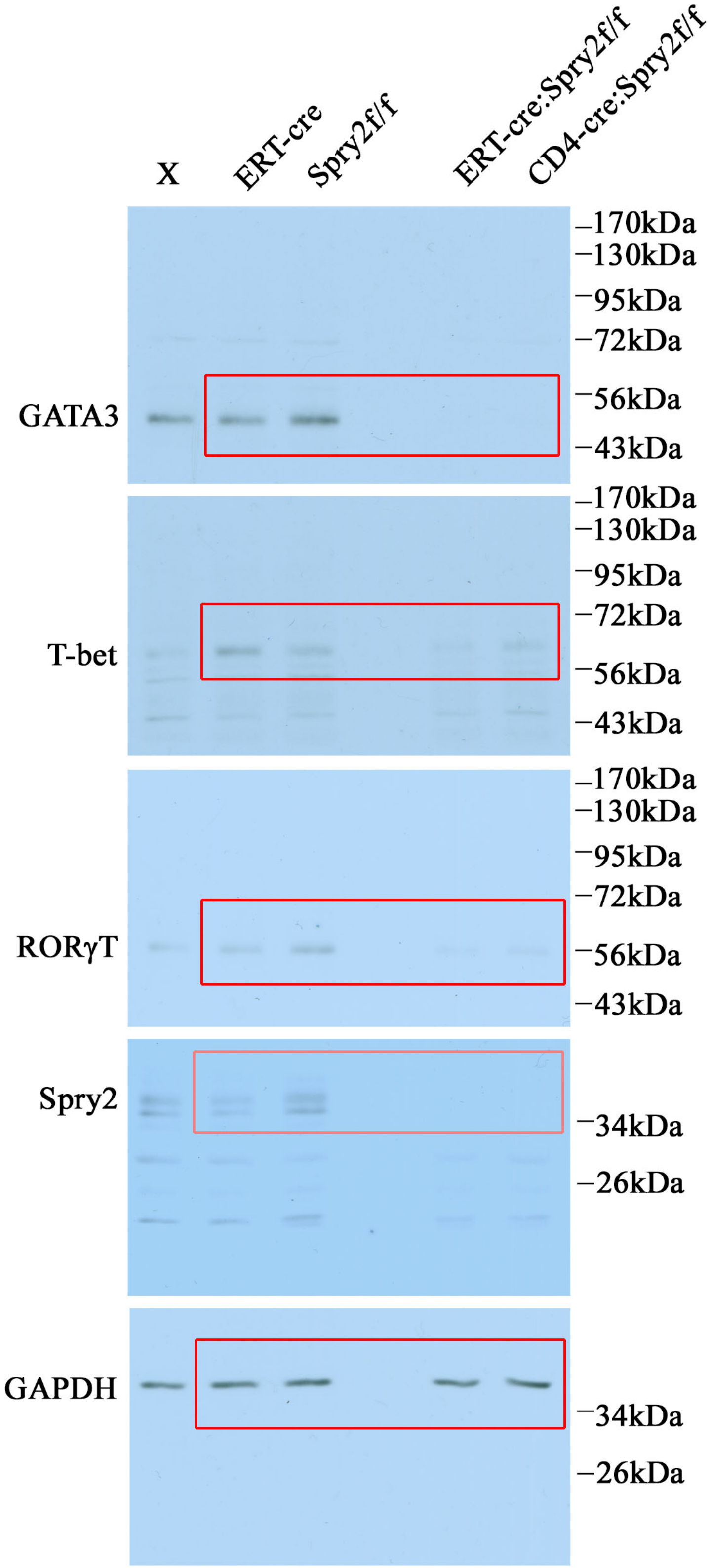

Full unedited Blot for Figure. 2 J

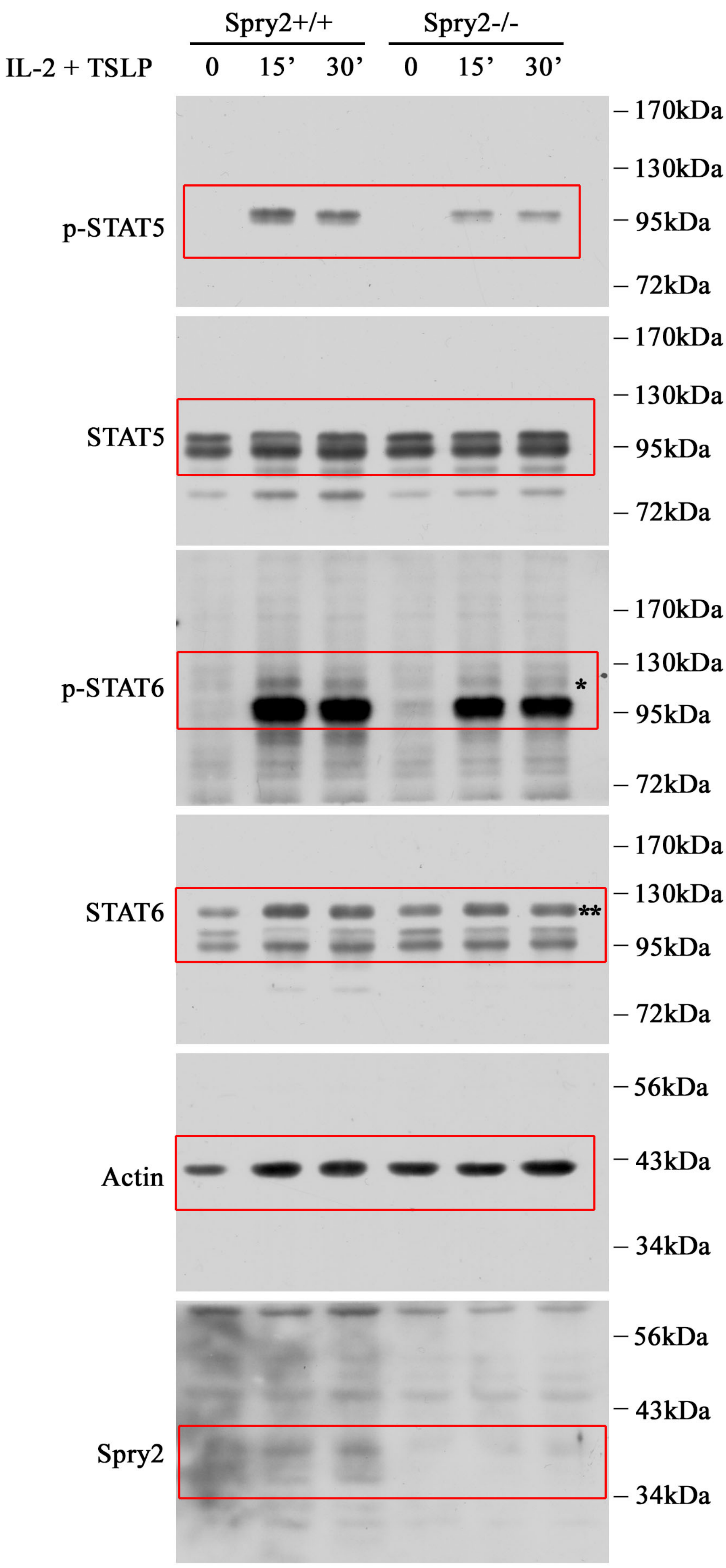

Full unedited Blot for Figure 6 A

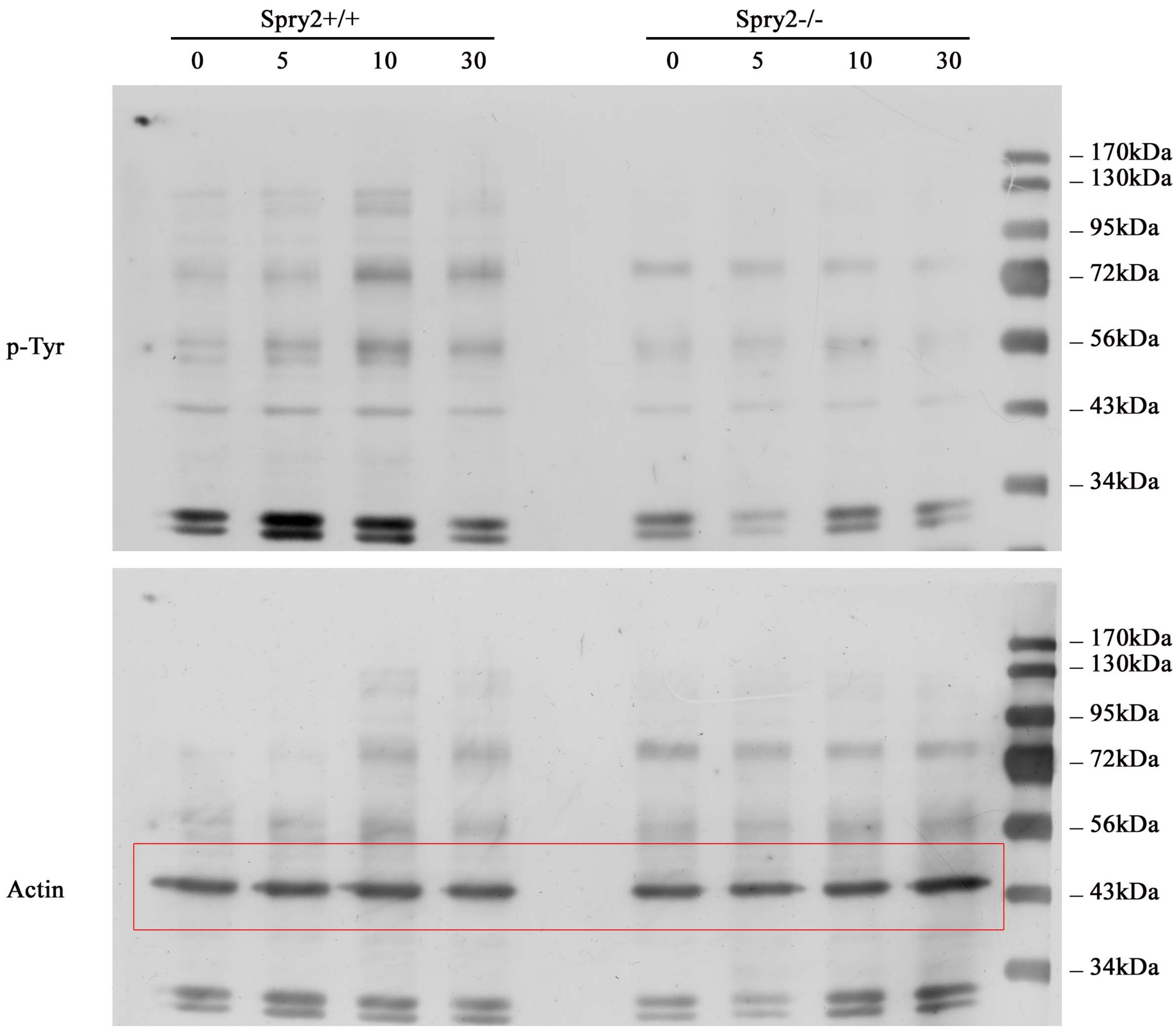

Full unedited Blot for Figure 6 B

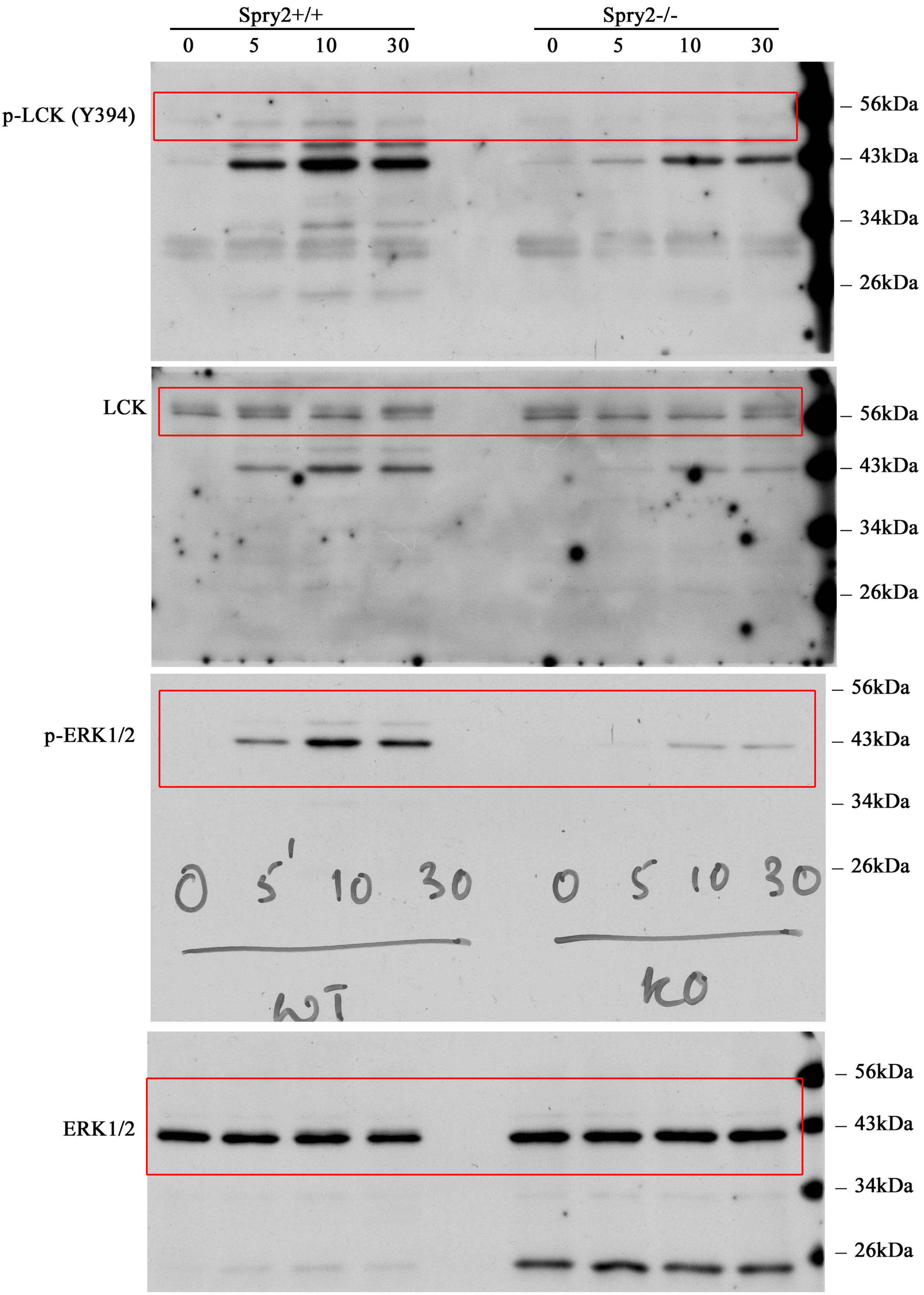

Full unedited Blot for Figure 6B (Cont'd)

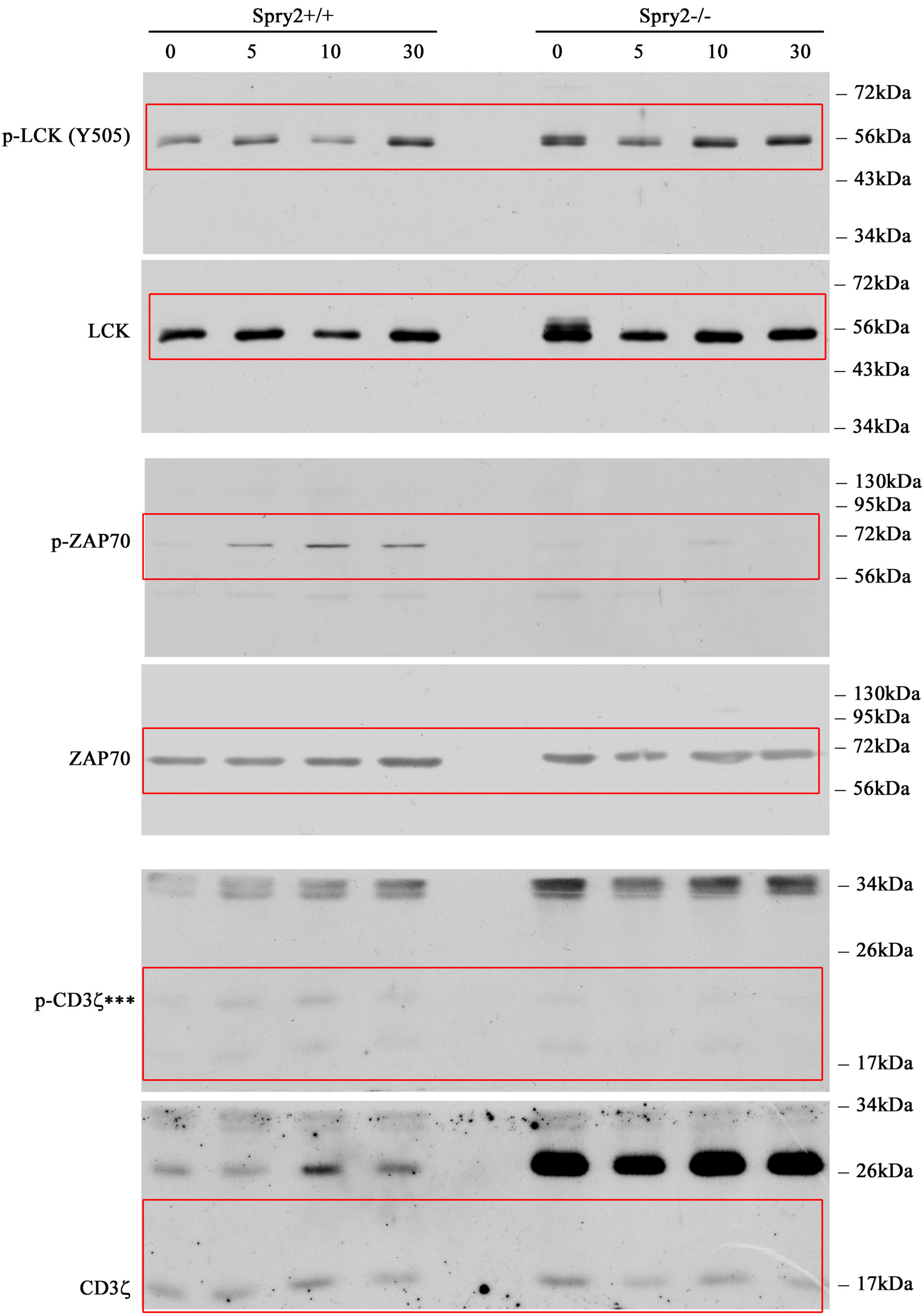

Full unedited Blot for Figure 6 F & G

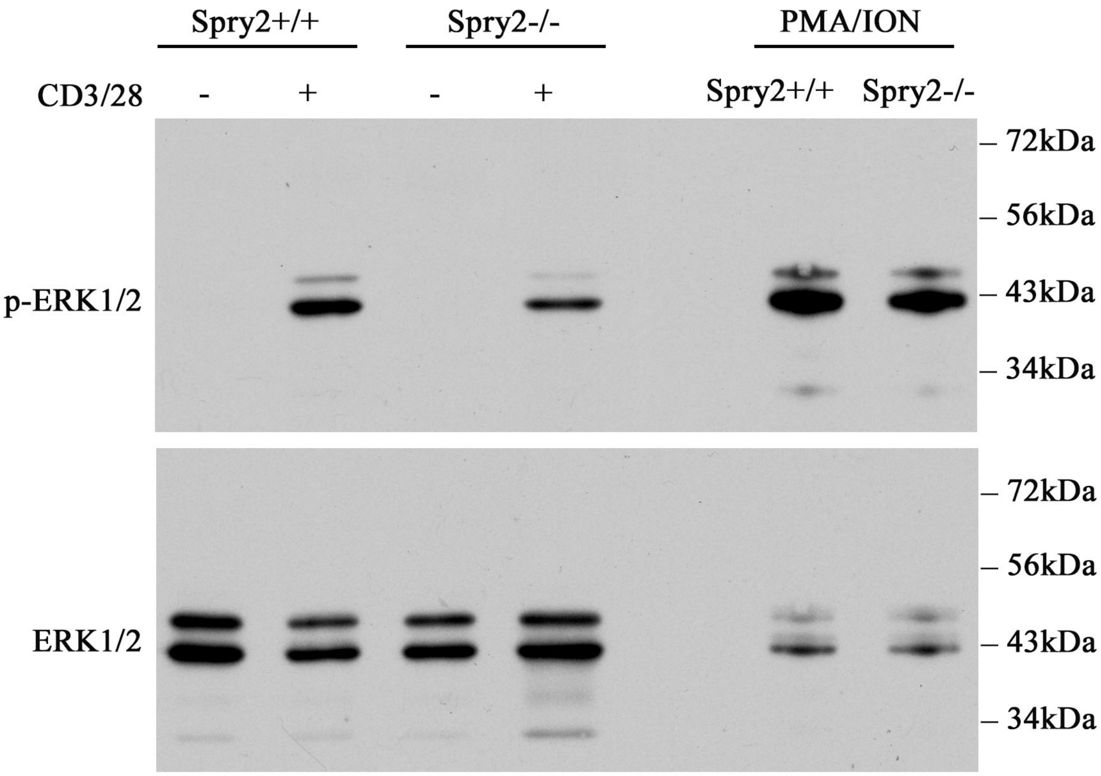

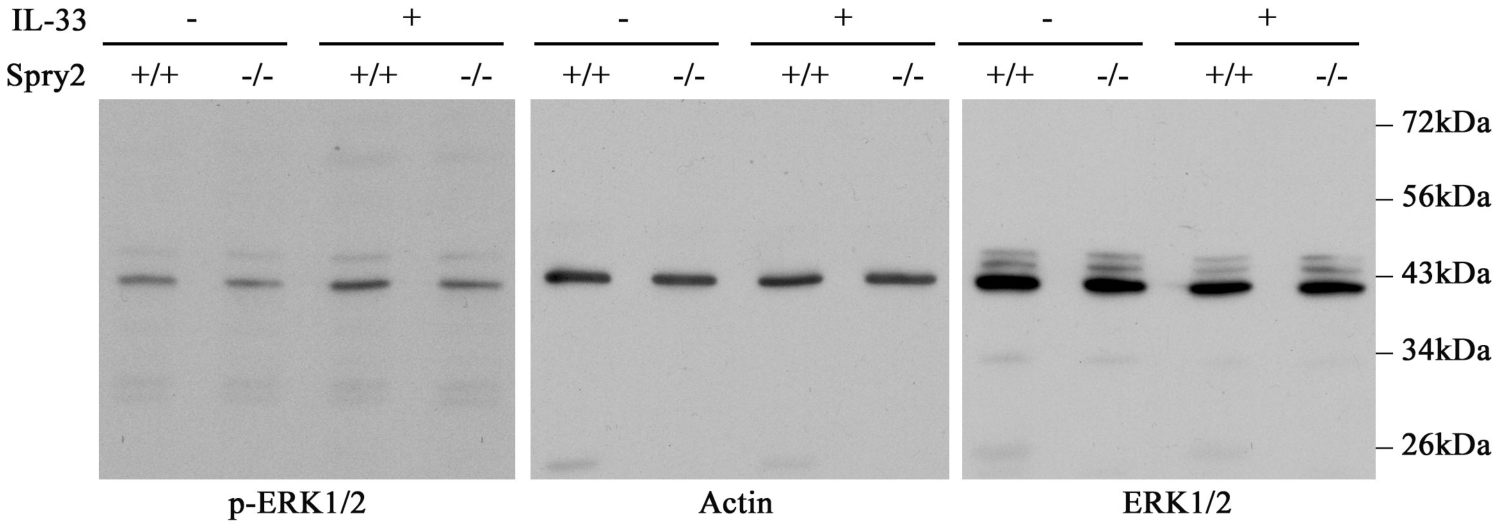

Full unedited Blot for Figure 7 C

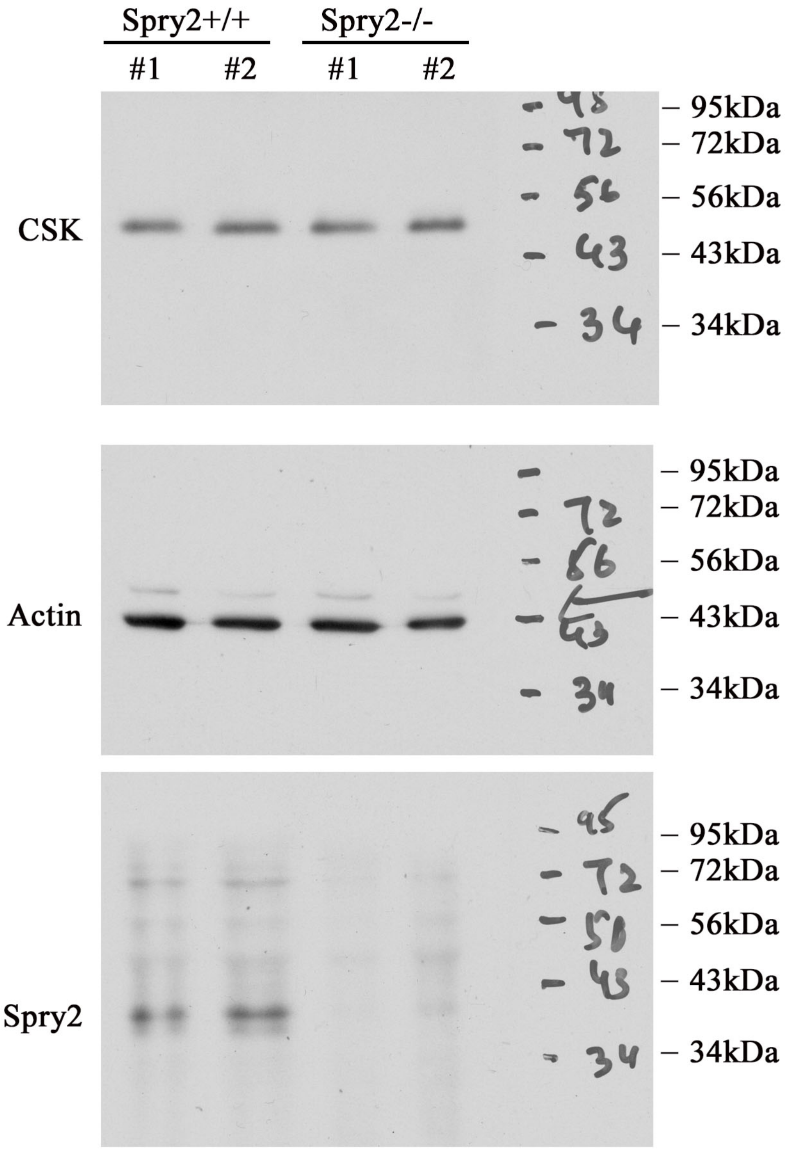

Full unedited Blot for Figure 7 D & E

Blots here were probed with ECL reagent and images are aquired using BioRad ChemiDoc

Figure 7 D

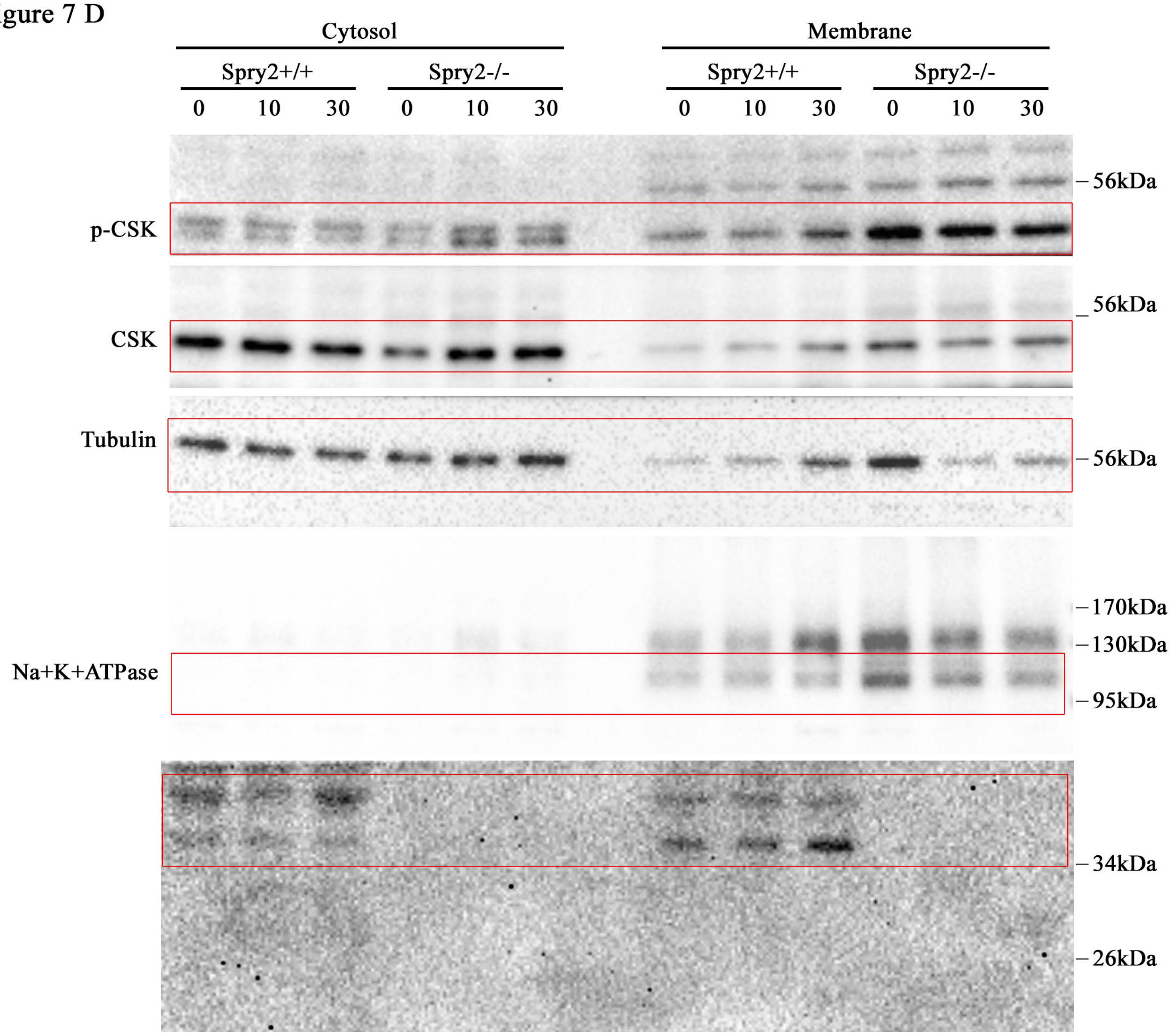

Figure 7 E

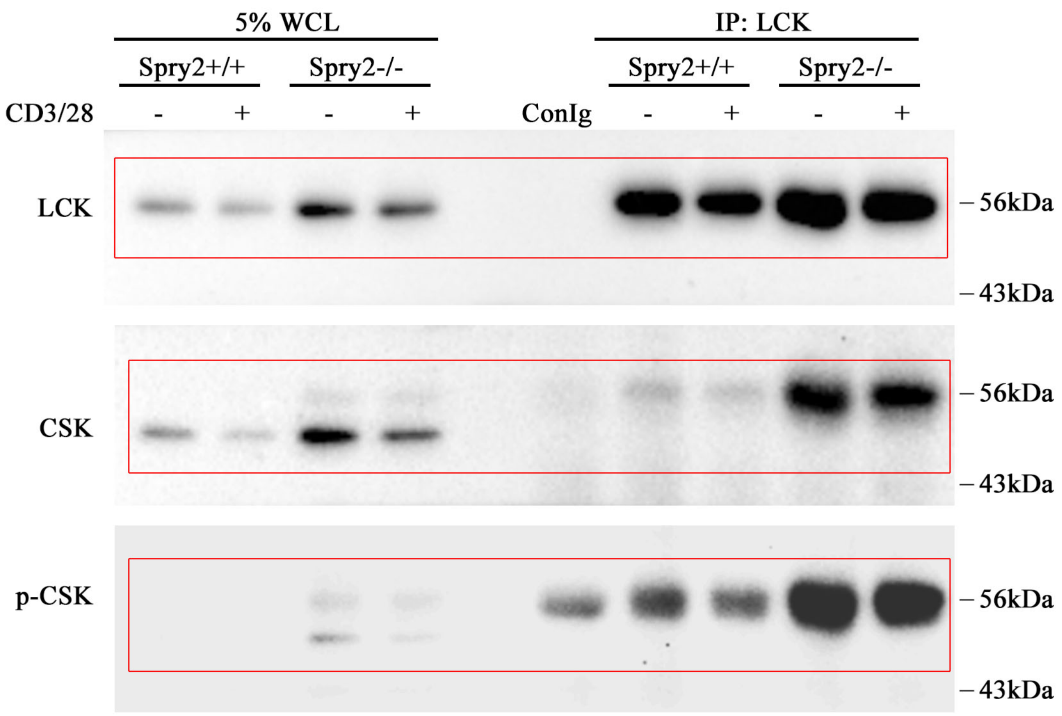

Full unedited Blot for Figure 8 A

Blots here were probed with ECL reagent and images are aquired using BioRad ChemiDoc

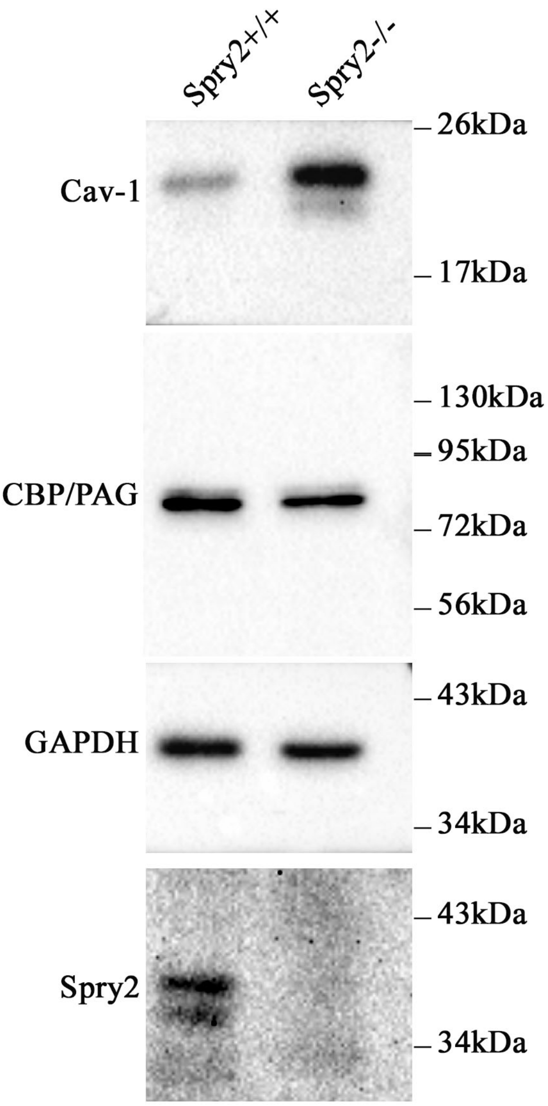

Full unedited Blot for Figure 8 B

Blots here were probed with ECL reagent and images are aquired using BioRad ChemiDoc

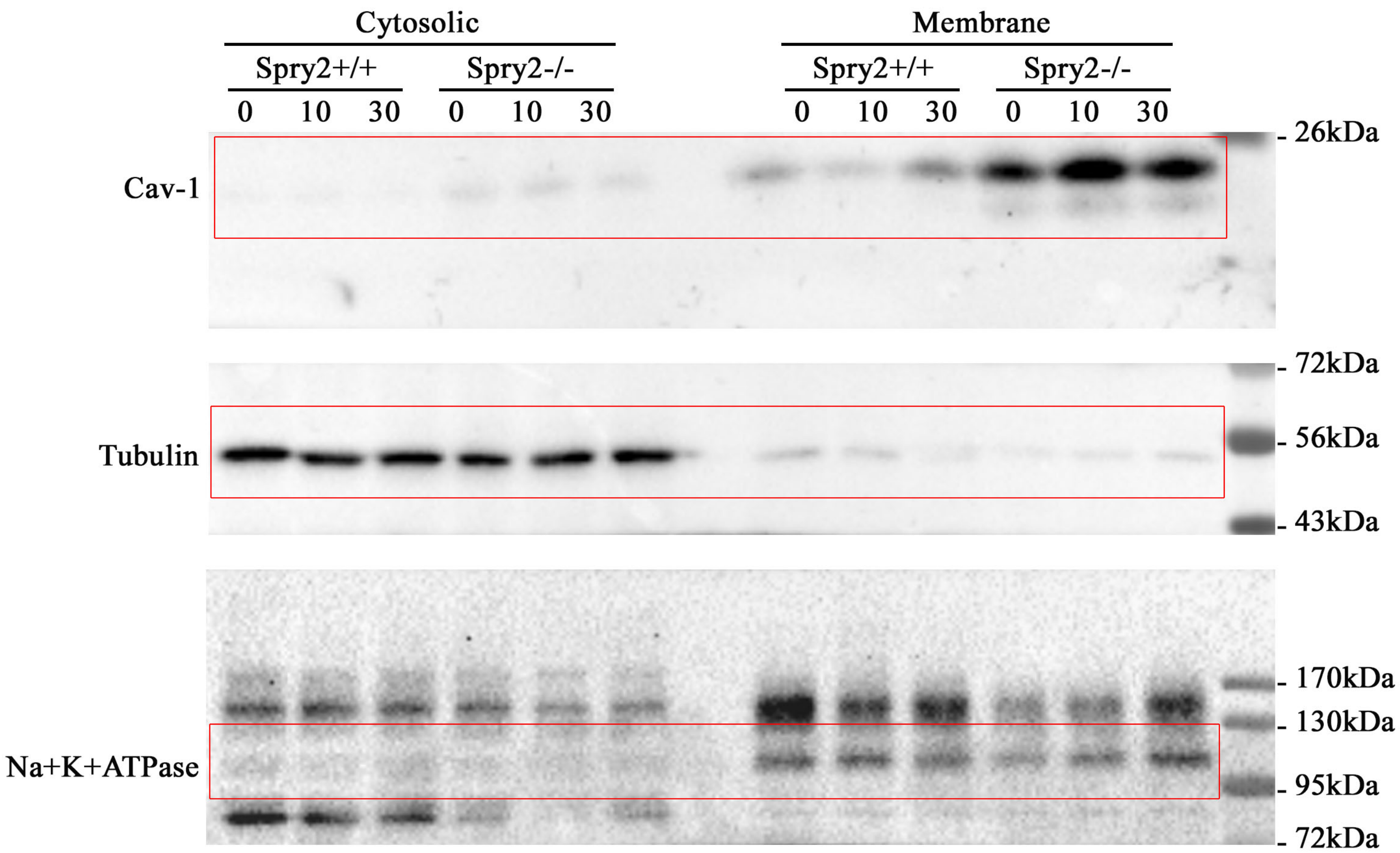

Full unedited Blot for Figure 8 G

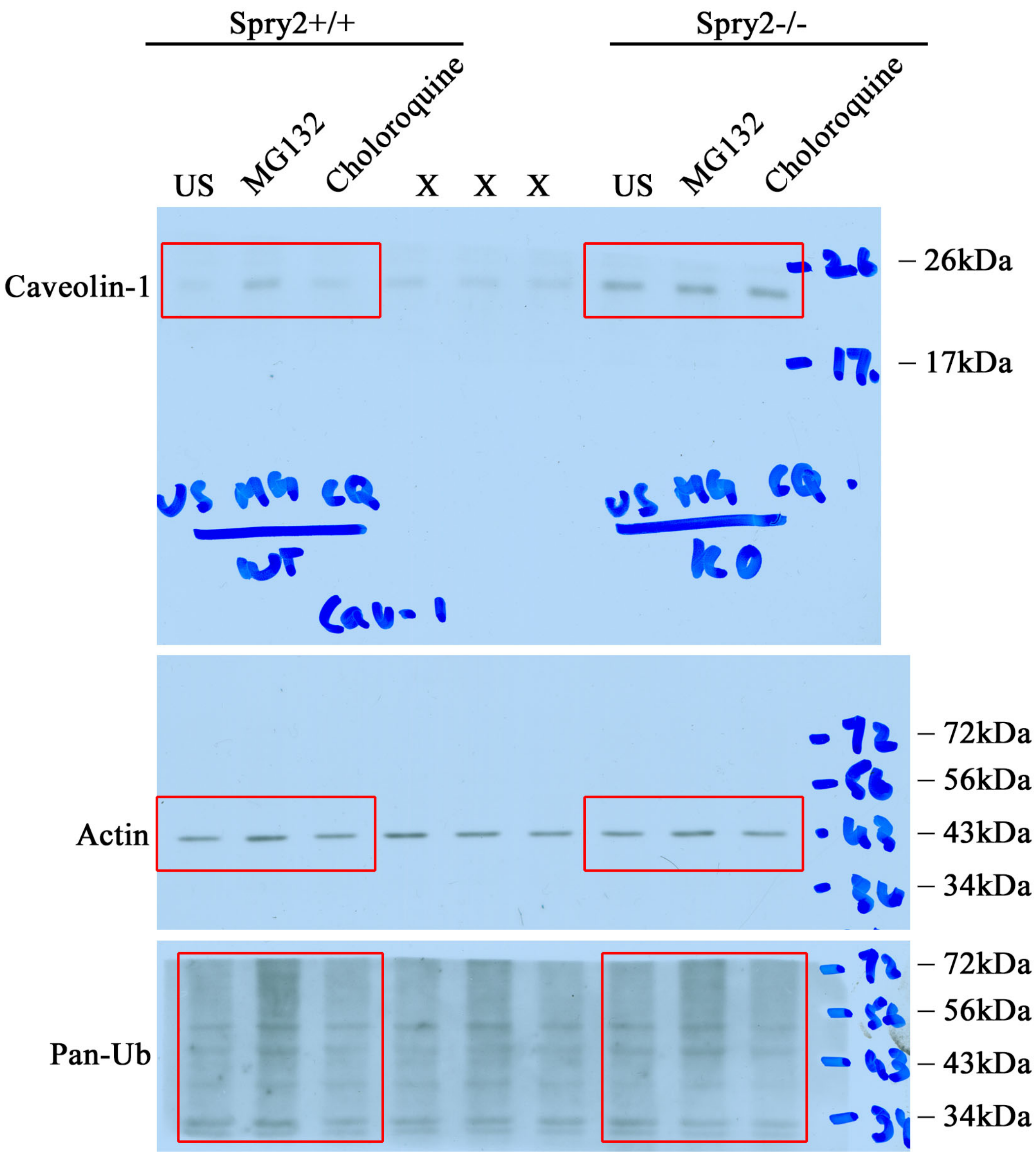

Full unedited Blot for Figure 8 H

Blots here were probed with ECL reagent and images are aquired using BioRad ChemiDoc

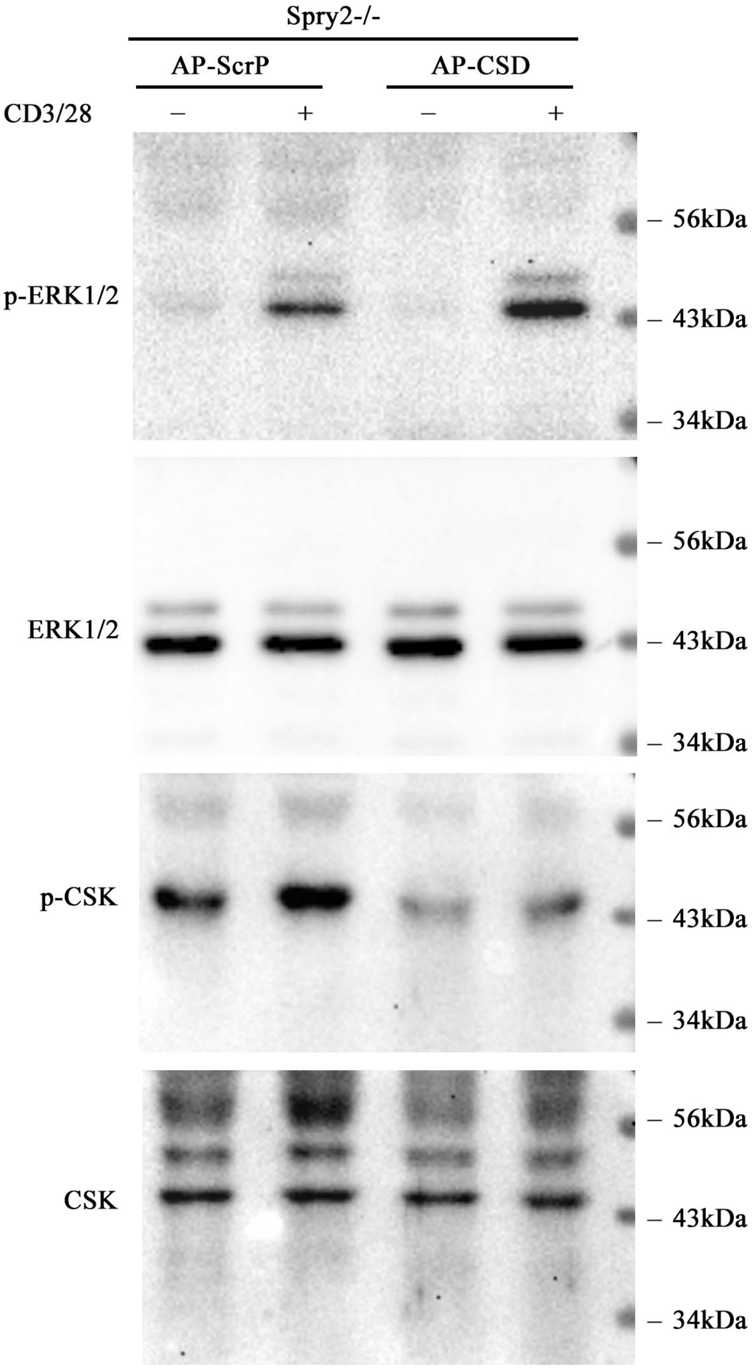

Full unedited Blot for Supplementary Figure 3A & B

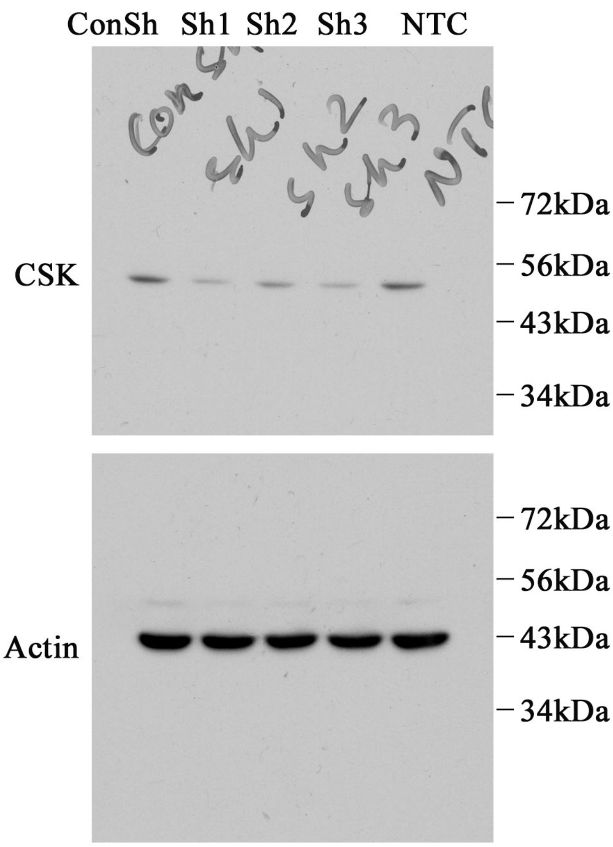

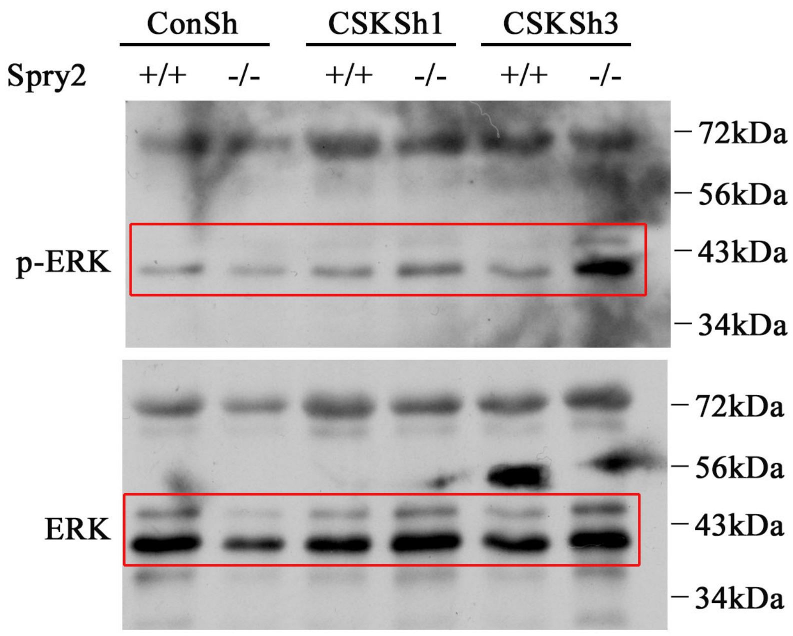

Full unedited Blot for Supplementary Figure 4 B

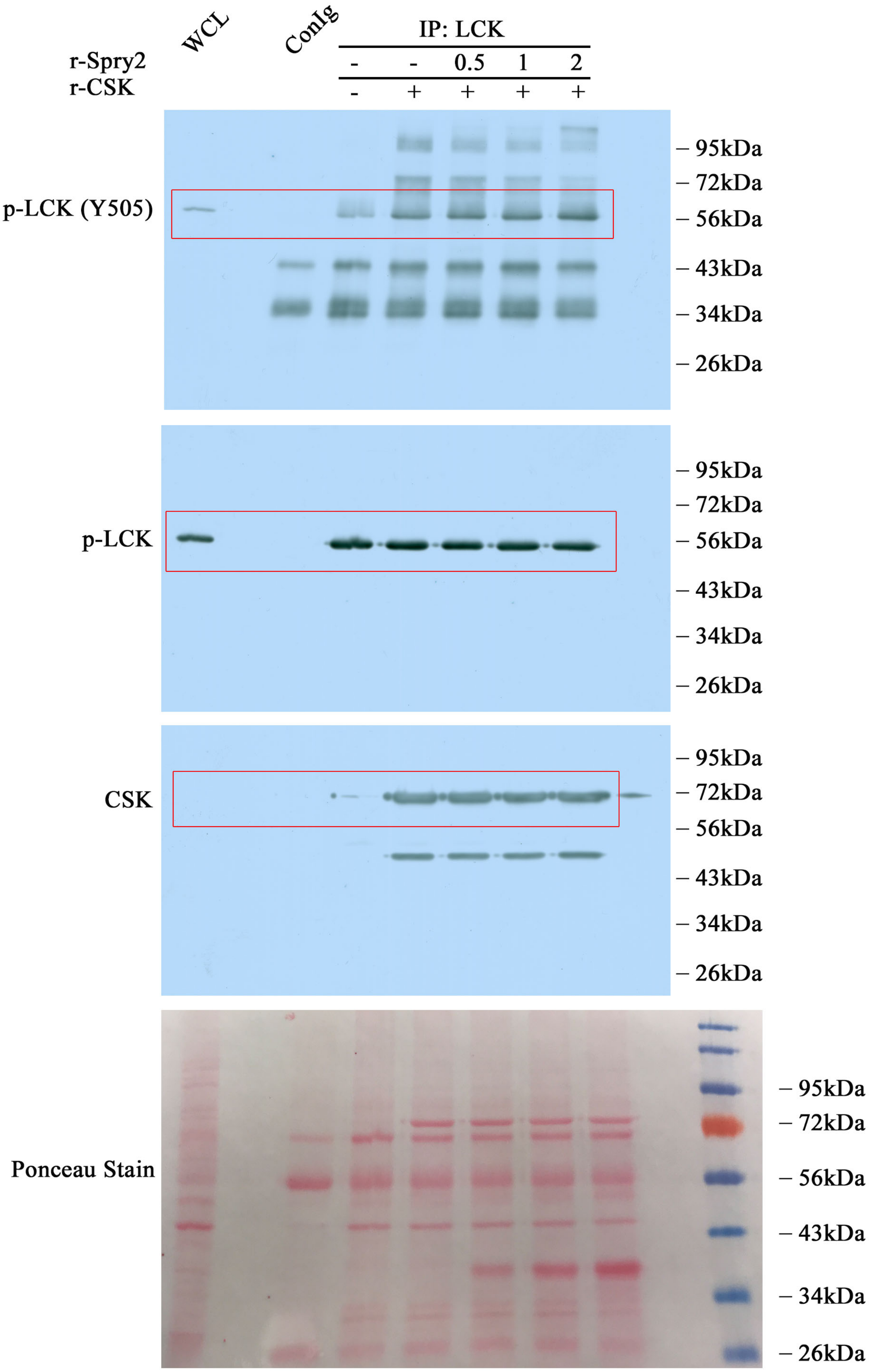

Fig 1C & E Gating strategy

Spleen

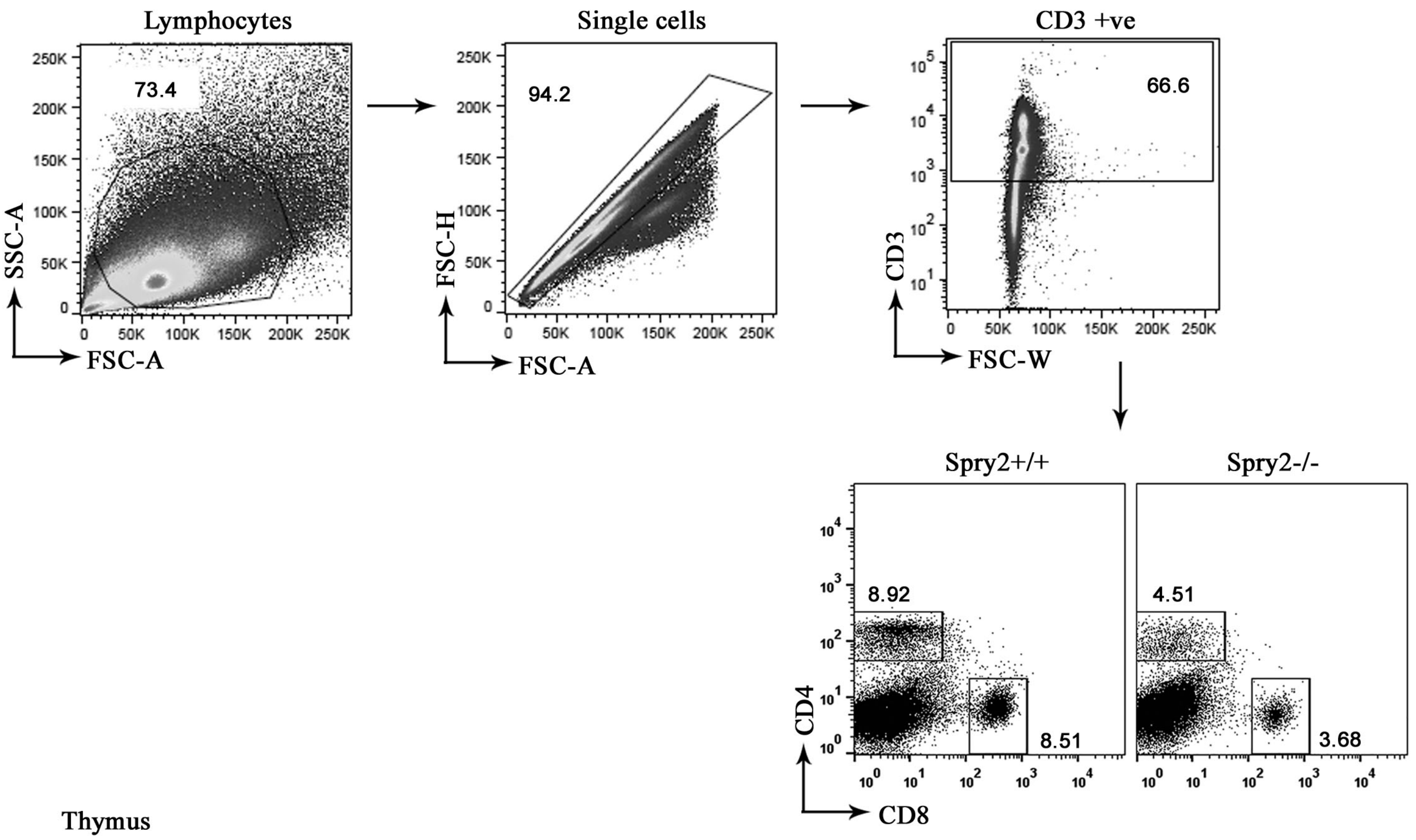

Thymus

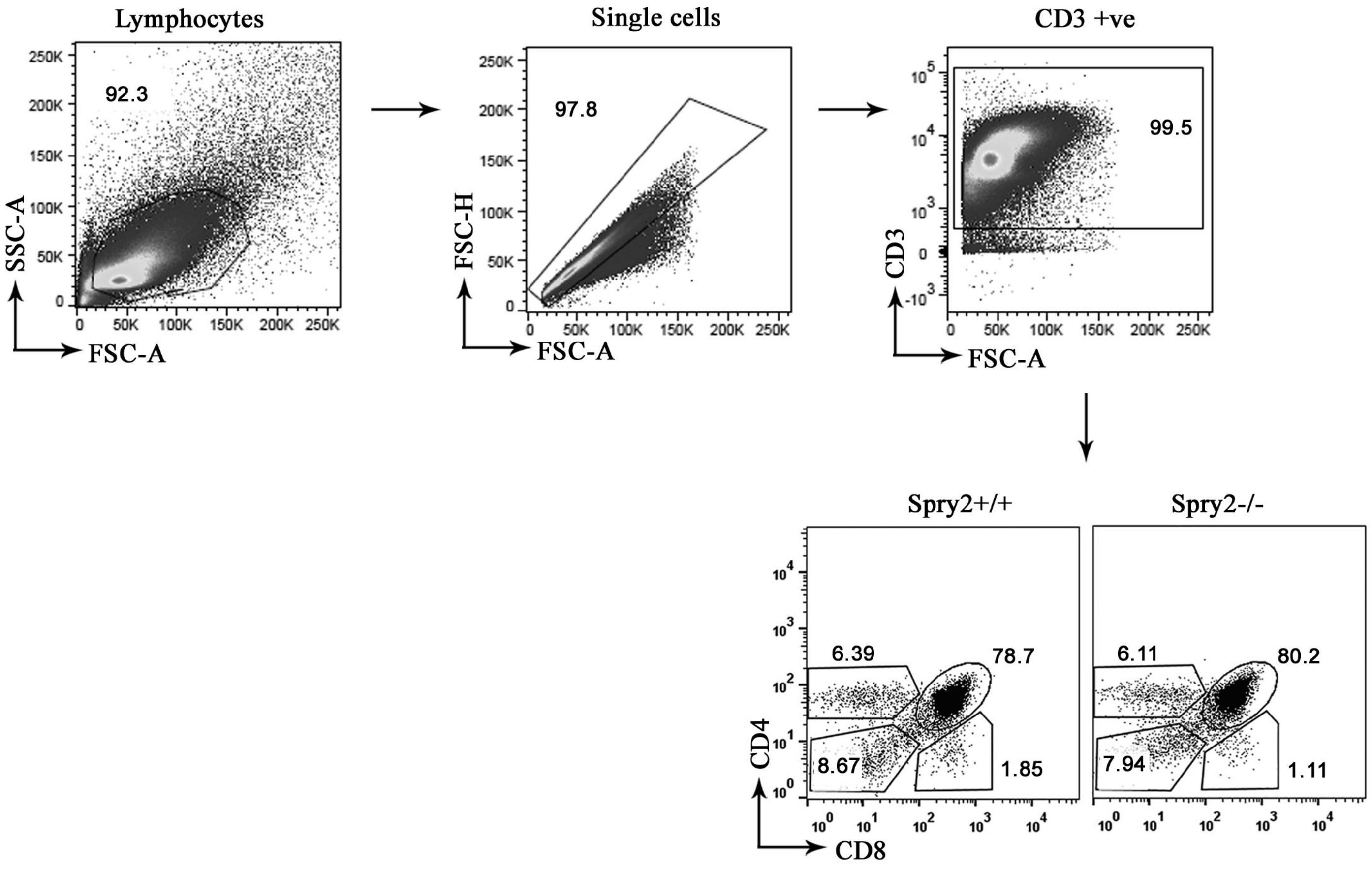

Fig 5A Gating strategy for CD4 T cells from Blood PBMCs and BAL

Blood PBMCs

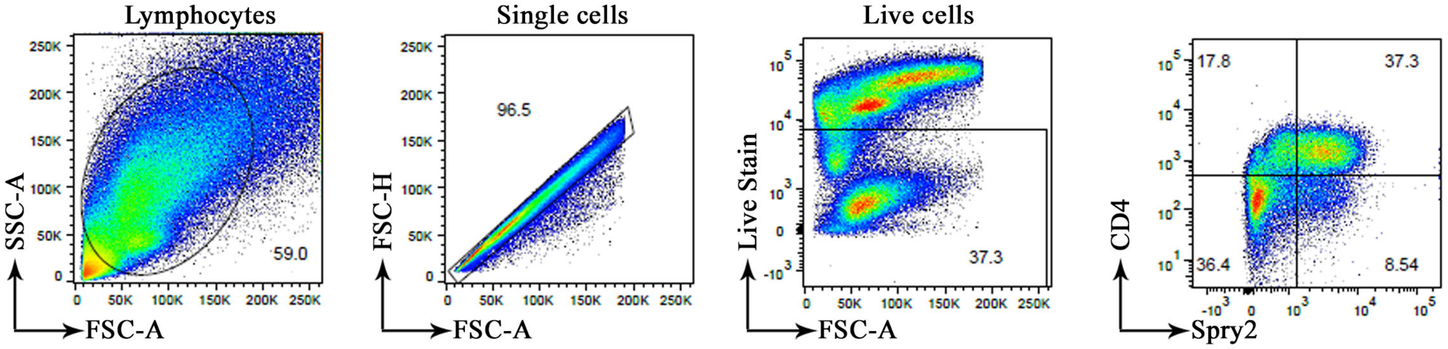

BAL

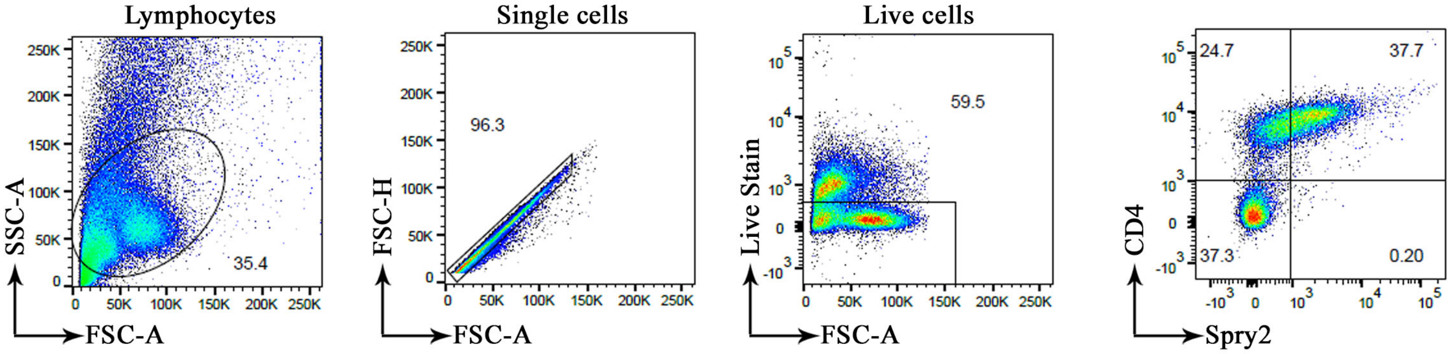

Supplement: S2 Data — Fig 1A. Western blot of spleen CD4+ T cells from Tamoxifen-treated Spry2f/f (Spry2+/+) and ERT2-Cre:Spry2f/f (Spry2−/−) mice for Spry2 and actin Fig 1H. Western blot showing Cleaved Caspase-3 (CC-3) levels in splenic CD4+ T cells from Spry2+/+ and Spry2−/− mice. Fig 2A. Immunoblot of basal Spry2 and GAPDH expression in thymocytes (Thy), splenocytes (Spl), naïve (Tn), and memory T cells from B6 mice. Fig 2C. Immunoblot of ERK activation, Spry2 protein induction, and actin expression in unstimulated (US) and stimulated (CD3 and CD3/28) human CD4+ T cells at indicated time points (24 h and 48 h, respectively); P/I represents phorbol myristate acetate and ionomycin. Fig 2G. Immunoblot of Spry2 and transcription factor (T-bet, GATA3, and RORgT) expression in naïve and differentiated CD4+ T cells from C57BL/6 mice (n = 3). Fig 2H. Immunoblot of T-bet, GATA3, and RORγT in CD4+ T cells from Spry2+/+ and Spry2−/− mice. Fig 2J. Immunoblot of p-STAT5 and p-STAT6 in CD4+ T cells, treated with IL-2 (10 ng/mL) and TSLP (10 ng/mL) at indicated time points (in min). * in the blot indicates the location of p-STAT6/STAT6 bands. Fig 6A. p-Tyr and β-Actin immunoblots of sort-purified, anti-CD3/CD28-stimulated CD4+ T cells from Spry2+/+ and Spry2−/− mice. Fig 6B. LCK, ZAP70, CD3ζ, and ERK immunoblots of sort-purified, anti-CD3/28-stimulated CD4+ T cells from Spry2+/+ and Spry2−/− mice. * indicates the location of p-CD3ζ bands. Fig 6F and 6G. p-ERK1/2 and ERK1/2 immunoblots of CD4+ T cells from Spry2+/+ and Spry2−/− mice stimulated with anti-CD3/CD28 or PMA/Ionomycin for 10 min (F) or recombinant IL-33 (20 ng/mL) for 30 min (G). Fig 7C. Immunoblot analysis of total CSK, actin, and Spry2 levels in Spry2+/+ and Spry2−/− CD4+ T cells. Fig 7D. p-CSK and CSK immunoblots of cytosol and membrane fractions of stimulated CD4+ T cells from Spry2+/+ and Spry2−/− mice. Na+ K+ ATPase and Tubulin serve as loading controls for membrane and cytosolic fractions, respectively. Fig 7E. Immunoprecipitation [file pbio.3001063.s004.pdf]
