# Supplementary figures and images for: Sprouty2 positively regulates T cell function and airway inflammation through regulation of CSK and LCK kinases
Source: PLoS Biol. 2021 Mar 8;19(3):e3001063. doi: 10.1371/journal.pbio.3001063 (PMC7971865; doi:10.1371/journal.pbio.3001063)

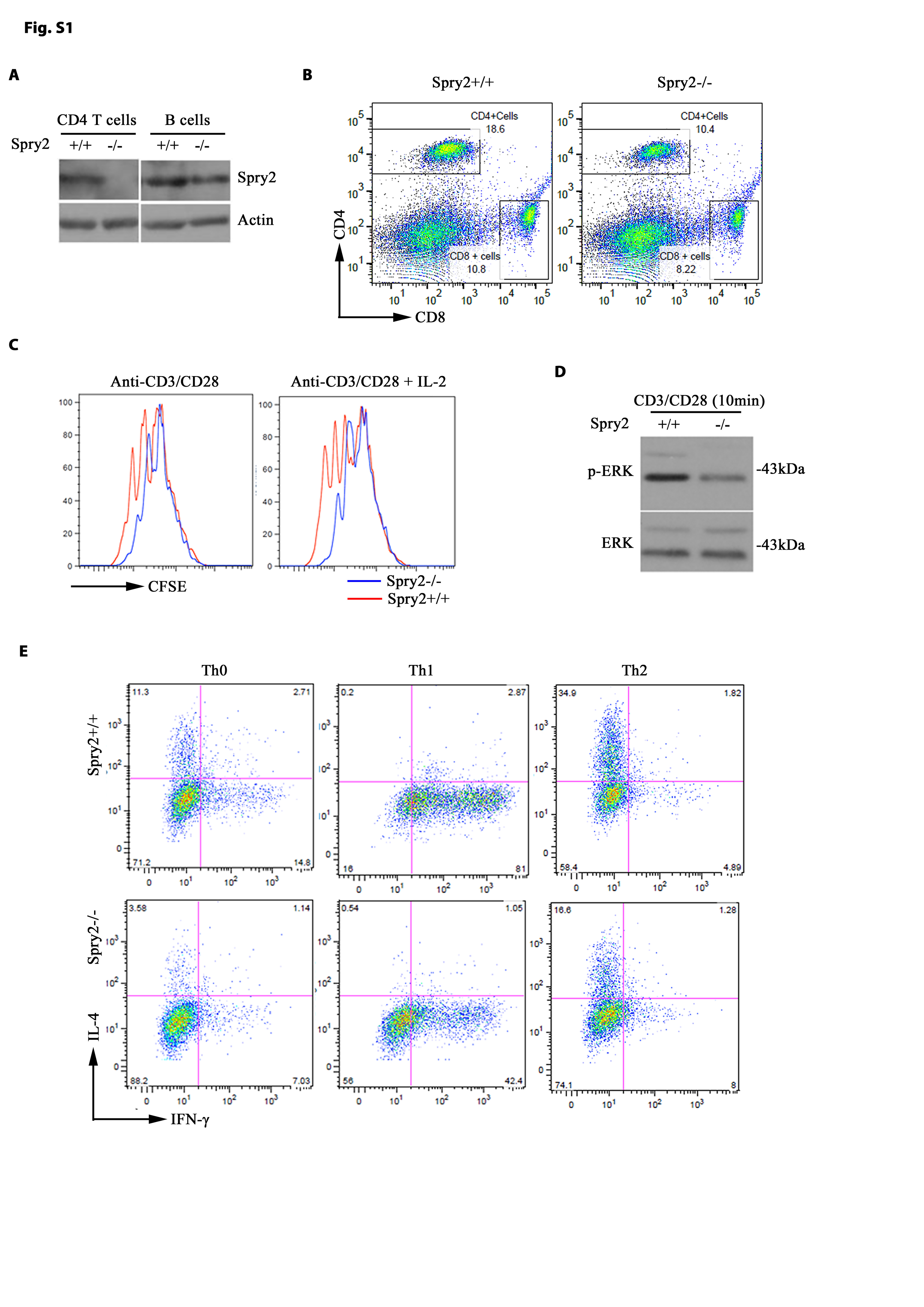

Supplement: S1 Fig — (A) Immunoblot analysis of CD4+ T cells and B cells from Spry2f/f and CD4-Cre:Spry2f/f mice (n = 2) confirm CD4+ T cell–specific deletion of Spry2. (B) Splenic CD4+ and CD8+ T cell frequencies from Spry2f/f and CD4-Cre:Spry2f/f mice (n = 3). (C) Proliferation of anti-CD3/CD28- or anti-CD3/28+IL-2–stimulated CD4+ T cells from Spry2f/f and CD4-Cre:Spry2f/f mice (n = 3). (D) Immunoblots of TCR-driven ERK1/2 phosphorylation in CD4+ T cells from Spry2f/f and CD4-Cre:Spry2f/f mice (n = 3). (E) Intracellular IFN-γ and IL-4 frequencies in CD4+ T cells from Spry2f/f and CD4-Cre:Spry2f/f mice under Th-skewing conditions (n = 3). (F) Representative images of H&E staining for lung tissue inflammation obtained from Sal or OVA-treated Spry2f/f and OVA-treated CD4-Cre:Spry2f/f mice. Scale bar, 100 μm. (G) Inflammatory area per μm of bronchial BM *** p < 0.0001 (n = 5 mice/group). All the data of this figure can be found in the S1 Data file. BM, basement membrane; ERK1/2, extracellular signal-regulated kinase 1/2; H&E, hematoxylin and eosin; IFN-γ, interferon gamma; OVA, ovalbumin; Sal, saline; Spry2, Sprouty2; TCR, T cell receptor. (TIF) [file pbio.3001063.s005.tif]

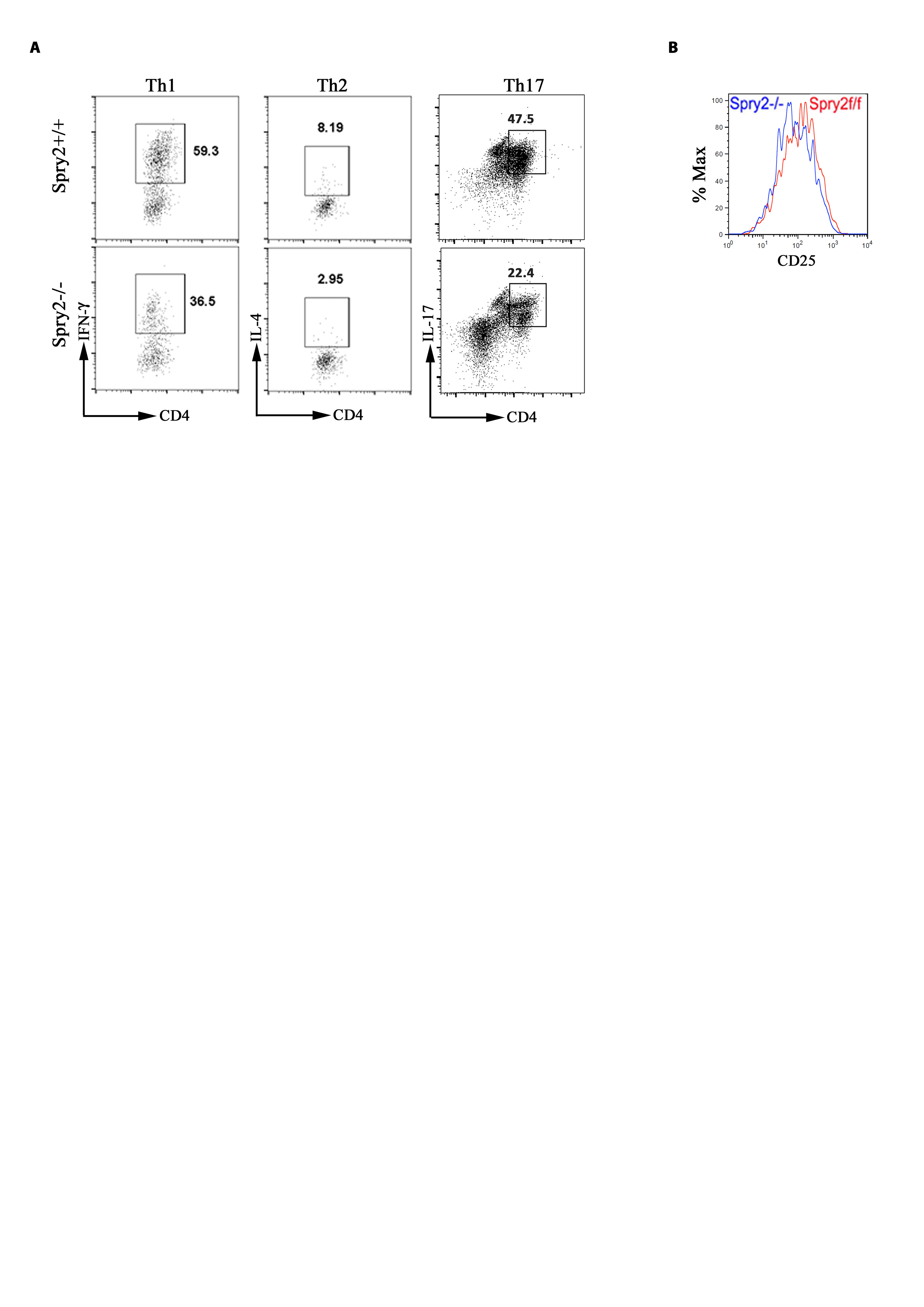

Supplement: S2 Fig — (A) Frequency of cytokine (IFN-γ, IL-4, and IL-17A)+ Spry2+/+ and Spry2−/− CD4 T cells cultured under Th1, Th2, and Th17 conditions as determined by flow cytometry (N = 3). (B) A flow cytogram depicting the surface expression of CD25 in splenic CD4+ T cells from Spry2+/+ and Spry2−/− mice. All the data of this figure can be found in the S1 Data file. IFN-γ, interferon gamma; Spry2, Sprouty2. (TIF) [file pbio.3001063.s006.tif]

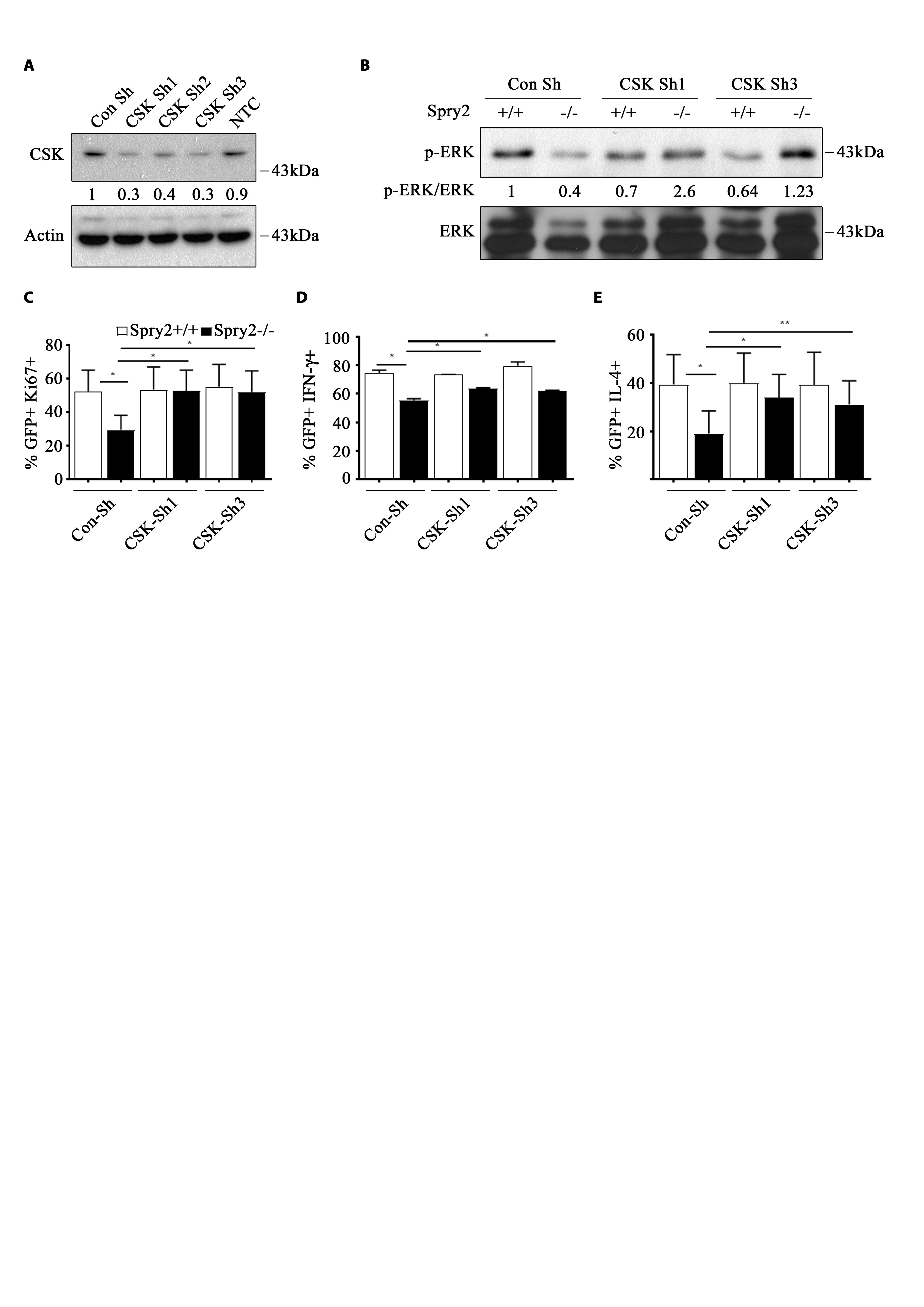

Supplement: S3 Fig — (A) An immunoblot showing lentiviral-mediated knockdown of endogenous CSK in CD4+ T cells from B6 mice. Numerical values indicate relative densitometric quantification of CSK normalized to actin. (B) Immunoblot of TCR-driven ERK1/2 phosphorylation in CD4+ T cells from Spry2+/+ and Spry2−/− mice transduced Con shRNA, CSK shRNA1, or CSK shRNA3. Numerical values indicate relative densitometric quantification of p-ERK1/2 normalized to total ERK1/2. (C–E) Proliferation as assessed by Ki67 staining (n = 3) and IFNγ (n = 3) and IL-4 secretion (n = 5) of GFP+ anti-CD3/CD28-stimulated (2 μg/mL each for 48 h) CD4+ T cells from Spry2+/+ and Spry2−/− mice transduced with GFP-expressing Con shRNA, CSK shRNA1, or CSK shRNA3. Significance * p < 0.05; **p < 0.005 by Student t test. All the data of this figure can be found in the S1 and S2 Data files. Con shRNA, Control shRNA; ERK1/2, extracellular signal-regulated kinase 1/2; GP, green fluorescent protein; IFN-γ, interferon gamma; NTC, nontransduced CD4+ T cells; TCR, T cell receptor. (TIF) [file pbio.3001063.s007.tif]

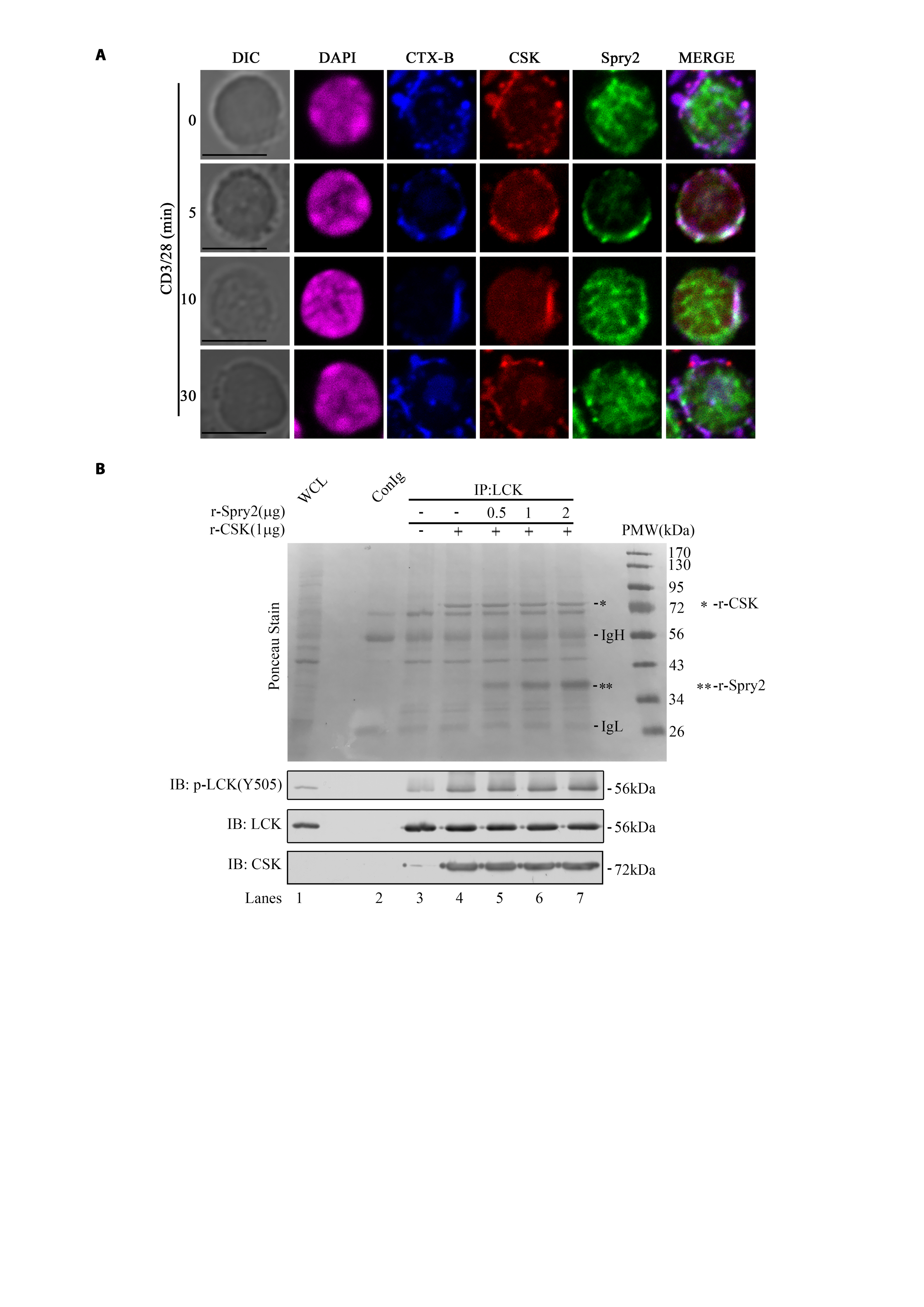

Supplement: S4 Fig — (A) Representative confocal images of CTX-B, CSK, and Spry2-stained, DAPI-counterstained anti-CD3/28-stimulated CD4+ T cells from B6 mice (n = 3). Scale bar, 5 μm. Colocalization shown in merged images. (B) Immunoprecipitation of LCK from murine CD4+ T cells followed by a kinase assay in the presence of r-CSK (Lanes: 4, 5, 6, 7 of the Ponceau stained blot) or r-Spry2 (Lanes: 5, 6, 7); WCL represents 5% total cell lysate; IgH and IgL represent Ig heavy and light chains, respectively. All the data of this figure can be found in the S2 Data file. CTX-B, Cholera Toxin-B; Ig, immunoglobulin; r-CSK, recombinant mouse CSK; r-Spry2, recombinant mouse Spry2; Spry2, Sprouty2; WCL, whole cell lysate. (TIF) [file pbio.3001063.s008.tif]
